# Unlocking Gd(III) Anisotropy: Determining the Zero-Field Splitting Axes to Enhance Spin-Label Structural Analysis

**DOI:** 10.1021/jacs.5c22555

**Published:** 2026-06-02

**Authors:** Alexey Bogdanov, Veronica Frydman, Xun-Cheng Su, Manas Seal, Wenkai Zhu, Angela M. Gronenborn, Alexander Schnegg, Daniella Goldfarb

**Affiliations:** † Department of Chemical and Biological Physics, 34976 The Weizmann Institute of Science, P.O. Box 26, Rehovot 7610001, Israel; ‡ Department of Chemical Research Support, The Weizmann Institute of Science, P.O. Box 26, Rehovot 7610001, Israel; § State Key Laboratory of Elemento-Organic Chemistry, 12538 Nankai University, Tianjin 300071, P. R. China; ∥ Department of Structural Biology, 6614 University of Pittsburgh, 4200 Fifth Ave, Pittsburgh, Pennsylvania 15260, United States; ⊥ 28313 Max Planck Institute for Chemical Energy Conversion, 34-36 Stiftstraße, Mülheim an der Ruhr 45470, Germany

## Abstract

The zero-field splitting (ZFS) of Gd­(III) complexes is central to magnetic resonance applications, influencing nuclear relaxation in MRI and NMR, electron spin relaxation in EPR, and the performance of Gd-based spin labels for structural biology applications. However, determining the molecular-frame orientation of the ZFS tensor is experimentally and computationally demanding for Gd­(III) chelate complexes in frozen solution, where structural and dynamic heterogeneity complicates orientation determination. Here, we introduce an experimental strategy that enables direct determination of the ZFS tensor orientation in Gd­(III) complexes using ^19^F and ^1^H orientation-selective (OS) electron–nuclear double resonance (ENDOR). For the commonly used spin labels Gd-DO3A and Gd-PyMTA, we determined the molecular-frame orientation of the ZFS, necessary for quantitative analysis of the Gd­(III) anisotropy. This allowed for a dual-mode analysis of Gd–F ENDOR spectra, where electron–nuclear distances can be accurately extracted from measurements at the Gd­(III) central transition, while the Gd–F vector orientation is determined using OS-ENDOR on high-|*m*_S_| transitions. Accordingly, we determined the location of Gd-DO3A labels in two ^19^F-containing proteins and showed that utilizing existing rotamer libraries substantially overestimates the label’s conformational distributions. The experimentally determined ZFS orientations are further compared to theoretical predictions of a simple molecular modeling calculation based on the electric-field-gradient tensor. Our results provide a platform for improved structure determination with Gd-based labels and offer valuable benchmarks for improving quantum-chemical predictions of ZFS tensors.

## Graphical abstract

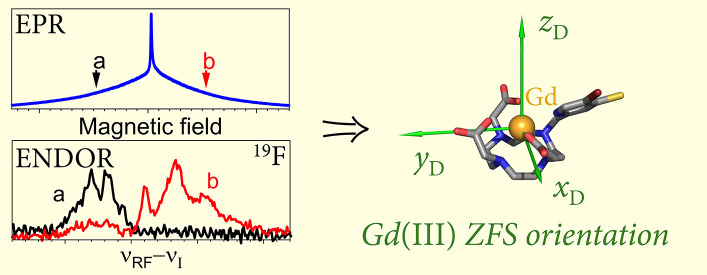

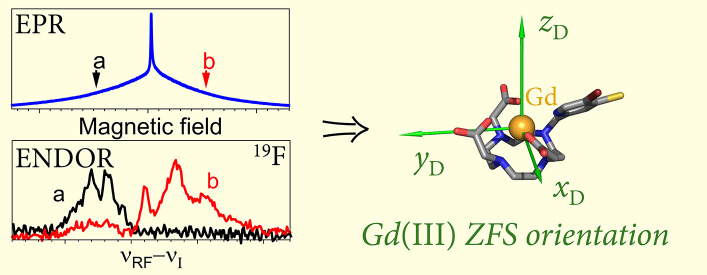

## Introduction

Gadolinium­(III) complexes play an important role in several key applications of magnetic resonance. They are used as spin labels for structural studies of biomacromolecules in solution and in cells by both NMR and EPR spectroscopies,^1–5^ help resolve structural features of host–guest systems,^6,7^ and act as efficient contrast agents in magnetic resonance imaging (MRI) owing to their influence on water proton relaxation.^8,9^ In addition, Gd­(III) complexes have been proposed as quantum hardware components, as their multiple low-energy states make them attractive for encoding qubits and qudits.^10,11^

The dominant anisotropic term in the spin Hamiltonian of Gd­(III) (S = 7/2) is the zero-field splitting (ZFS), which describes the splitting of the electronic spin energy levels in the absence of an external magnetic field. The ZFS arises from spin–spin interactions between the unpaired electrons and from spin–orbit coupling, particularly in low-symmetry environments possessing high electrostatic anisotropy. In Gd­(III), which has a half-filled 4f shell, first-order spin–orbit coupling is absent, and ligand-field effects are weak. Thus, ZFS in Gd­(III) originates only from second-order spin–orbit coupling and small distortions in low-symmetry environments.^12^ The magnitude and fluctuations of Gd­(III) ZFS are critical, as they reflect the local structure of the Gd­(III) environment, govern nuclear relaxation,^13^ and influence the spin relaxation properties of Gd­(III) itself,^14,15^ thereby determining the efficiency with which structural information can be extracted from Gd­(III)-based spin labels. Accurate prediction of ZFS from an atomic structure would therefore enable the rational design of optimized Gd­(III) complexes for specific applications.

The ZFS Hamiltonian is given by

1 $${Ĥ}_{\mathrm{ZFS}}=D\left[{Ŝ}_{z}^{2}-\frac{1}{3}S\left(S+1\right)\right]+E\left({Ŝ}_{x}^{2}-{Ŝ}_{y}^{2}\right)$$

where *D* and *E* are related to the principal components of the ZFS tensor:

2 $$D=\frac{3}{2}D_{\mathrm{zz}},,E=\frac{1}{2}\left(D_{xx}-D_{yy}\right),,\mathrm{and}\;D_{xx}+D_{yy}+D_{zz}=0$$

The parameters *D* and *E* quantify the ZFS axial and rhombic anisotropies, respectively.

Because of its electronic structure with a half-filled 4f shell, Gd­(III) is outstanding in the lanthanides series. Direct measurements of the magnetic susceptibility of Gd­(III) using single-crystal torque magnetometry show that it possesses very small anisotropy,^16^ consistent with its ZFS originating from only second-order effects. Nevertheless, despite the small Gd­(III) ZFS, its signature is clearly evident in EPR spectra, and it can, in principle, be extracted from the spectra. For single crystals, one can determine both the magnitude (*D* and *E*) and the orientation of the ZFS tensor principal axis system (PAS) in the molecular frame.^17,18^ These measurements are typically done in magnetically diluted samples to prevent the unwanted effects of intermolecular spin–spin interactions on spin relaxation and spectral shapes.

In MRI and bioNMR studies, Gd­(III) typically exists in solution, where orientational disorder precludes the direct determination of the ZFS orientation. In frozen solutions, EPR spectra can provide only the values of *D* and *E*. Complicating matters further, unlike in crystals, the ZFS parameters of Gd­(III) complexes in frozen solutions exhibit broad distributions because of the presence of small variations in coordination bond lengths and angles.^19,20^ This often prevents a unique determination of *D* and *E*, necessitating measurements at multiple frequencies and/or temperatures to reduce uncertainty.^20^ Moreover, the transition from crystalline to glassy state has been shown to cause significant changes in ZFS principal components and may change the sign of *D*.^13^ Crucially, this leaves the orientation of the ZFS tensor within the molecular frame unknown.

Considerable efforts have been devoted to quantum-chemical calculations of Gd­(III) ZFS, particularly in the context of MRI contrast agents.^12,21–23^ Yet, its small magnitude and the dependence on approximations render the prediction of experimental *D* and *E* values challenging. Post-Hartree–Fock methods, such as CASSCF, provide more reliable static ZFS values than those obtained from DFT, and they generally capture the correct order of magnitude.^21^ In this context, providing accurate, experimentally determined orientations of the ZFS in the molecular frame offers valuable benchmarks for refining theoretical approaches for predicting both the magnitude and orientation of ZFS.

In this work, we present a methodology based on ^19^F electron–nuclear double resonance (ENDOR) of Gd­(III) spin label derivatives that enables the experimental determination of the Gd­(III) ZFS PAS in the molecular frame. We then demonstrate the use of the PAS in elucidating the structure of Gd­(III)-labeled proteins beyond Gd–F distances by determining the orientation of the Gd–F vector relative to the geometrical structure of the Gd­(III) label, thereby providing experimental constraints on the conformational ensemble.

Recently, the application of Gd­(III) spin labels for providing structural information on fluorine-containing proteins via Gd­(III)–^19^F distances has been demonstrated, using W-band ENDOR, both in frozen solutions and in cells^5,24^ A key spectroscopic advantage of Gd­(III) is its intense central transition (CT, *m*_S_ = −1/2 ↔ *m*_S_ = +1/2), the width of which is determined by second-order contributions of the ZFS, rendering it nearly isotropic over the ZFS ranges typical for Gd­(III) spin labels. Accordingly, an ENDOR measurement at a single magnetic field within the CT suffices to determine the Gd–F distance.^5,24^ This contrasts with nitroxide spin labels, where the EPR spectrum is dominated by the anisotropic Zeeman and ^14^N-hyperfine interactions,^25^ and ENDOR measurements at multiple fields within the EPR spectrum are required for distance determination. This approach, referred to as orientation-selection ENDOR (OS-ENDOR), is a well-established method for determining both distances and orientations of the electron–nuclear vector relative to the g-tensor principal axes.^26,27^ This gives useful structural information when the anisotropy is determined by the g-tensor, the orientation of which within the paramagnetic center is usually known, like in the case of nitroxides. Analysis of such sets of OS-ENDOR spectra, although tedious, provides not only the distance but also the orientation of the NO–^19^F vector relative to the g-tensor principal frame.^25,28^

For Gd­(III), OS-ENDOR has long been considered impractical due to the isotropic character of the CT and the large ZFS distributions, which yield broad, featureless spectra for the off-CT transitions. Exceptions are Gd­(III) centers with unusually large ZFS, where orientation selection can be observed even at the CT.^7,29^ Recently, we have shown that the resolution of ^19^F ENDOR of Gd­(III)-labeled proteins^30^ can be enhanced by low-temperature measurements targeting transitions other than the CT.^31^ Unexpectedly, we also observed clear orientation selection, despite the featureless line shape of the noncentral transitions and the broad distribution of the ZFS parameters *D* and *E*. This observation constitutes the foundation for the present work, in which we rationalize the observed orientation selection and use it to experimentally determine the orientation of the ZFS PAS for two Gd­(III) complexes that serve as chelating cores in commonly used spin labels. With this knowledge, we applied the OS-ENDOR approach to determine the positions of the Gd­(III) spin label with respect to the ^19^F atoms in a protein. Finally, we performed *ab initio* molecular dynamics (AIMD) calculations and demonstrated that the experimentally obtained ZFS orientations can be reasonably well predicted by the electrostatic-field gradient around the Gd­(III) ion.

## Theoretical Background

The Gd­(III) static spin Hamiltonian considered in this work is

3 $${Ĥ}_{0}={Ĥ}_{\mathrm{Zeeman}}+{Ĥ}_{\mathrm{ZFS}}+{Ĥ}_{n}$$

The first term describes the electron Zeeman interaction, the second describes the ZFS given by eq 1, and the third, ${Ĥ}_{n}$ , describes the hyperfine and nuclear Zeeman interactions. When the EPR spectrum is considered, only the first two terms in eq 3 are important, as ${Ĥ}_{n}$ is smaller than the EPR spectral resolution. In this case,

4 $${Ĥ}_{\mathrm{EPR}}=gB\cdot Ŝ+D\left[{Ŝ}_{z}^{2}-\frac{1}{3}S\left(S+1\right)\right]+E\left({Ŝ}_{x}^{2}-{Ŝ}_{y}^{2}\right)$$

where ${Ĥ}_{\mathrm{EPR}}$ is described in the ZFS PAS. The orientation of the magnetic field **B**, with the strength *B*_0_, in the ZFS frame is given by the polar angle θ_0_ and the azimuthal angle φ_0_ (Figure S1) and

5 $$B_{x}=B_{0}\sin\theta_{0}\cos\varphi_{0},,B_{y}=B_{0}\sin\theta_{0}\sin\varphi_{0},,B_{z}=B_{0}\cos\theta_{0}$$

The nuclear spin Hamiltonian, ${Ĥ}_{n}$ , relevant to the ENDOR spectrum, is given by

6 $${Ĥ}_{n}=g_{n}B\cdot Î+Ŝ\cdot A\cdot Î$$

Here, **A** is the hyperfine tensor, which in our case is dominated by the anisotropic electron–nuclear dipolar interaction, with potentially a small contribution from the isotropic part *a*_iso_. The total hyperfine coupling is given by

7 $$a\left(\beta \right)=\left(3\cos^{2}\beta -1\right)\frac{\mu_{0}g_{e}\mu_{B}g_{n}\mu_{N}}{4\pi hr^{3}}+a_{\mathrm{iso}}=\left(3\cos^{2}\beta -1\right)\left|a_{⊥}\right|+a_{iso}$$

where μ_0_ is the vacuum magnetic permeability, *g*_e_ and *g*_n_ are the electron and nuclear g-values, μ_B_ and μ_N_ are the Bohr magneton and nuclear magneton, respectively, *h* is the Planck constant, and *r* is the Gd–F distance. β is the angle between the external magnetic field **B** and the Gd–F vector, and *a*_⊥_ represents the dipolar hyperfine coupling when the magntic field is perpendicular to the Gd−F vector (β = π/2). The Gd–F vector is described in the PAS of the ZFS tensor by the polar and azimuthal angles γ and ρ, respectively (cf. Figure S1)­

8 $$\cos\beta =\cos\rho \sin\gamma \cos\varphi_{0}\sin\theta_{0}+\sin\rho \sin\gamma \sin\varphi_{0}\sin\theta_{0}+\cos\gamma \cos\theta_{0}$$

The ENDOR resonance frequencies for allowed NMR transitions (|Δ*m*_I_| = 1) at a high field, with relatively small hyperfine coupling and small ZFS, are given by^32^

9 $$\nu \left(\beta ,m_{S}\right)=\nu_{I}-m_{S}\cdot a\left(\beta \right)$$

Even though the first-order spin–orbit coupling in Gd­(III) is absent and the ZFS is not directly proportional to the crystal field splitting, the superposition approximation of Newman and Urban states that, the symmetry and the principal axes of the ZFS are governed by the charge distribution created by the ligands around the Gd­(III) ion.^33^ This approach relates the ZFS and the electric field gradient (EFG) tensorial components. From the point charge distribution, the electrostatic potential, ϕ, is given by calculated according to

10 $$\phi =\underset{i}{\sum }\frac{1}{4\pi \varepsilon_{0}}\cdot \frac{q_{i}}{r_{i}}$$

where *q*_*i*_ is the charge on the *i-*th atom and *r*_*i*_ is its distance from the central ion. The EFG is obtained from the second derivatives of ϕ:

11 $$V_{\alpha \beta }=\frac{\partial^{2}\phi }{\partial r_{\alpha }\partial r_{\beta }}=-\frac{\partial F_{\alpha }}{\partial r_{\beta }}=\underset{i}{\sum }\frac{q_{i}}{4\pi \varepsilon_{0}}\cdot \frac{3r_{\alpha ,i}r_{\beta ,i}-\delta_{\alpha \beta }r_{i}^{2}}{r_{i}^{5}}$$

where $r_{\alpha (\beta ),i}=x_{i},y_{i},z_{i}$ are the Cartesian coordinates of the *i*-th atom, with $r_{i}^{2}=x_{i}^{2}+y_{i}^{2}+z_{i}^{2}$ . $F_{\alpha }=-\underset{i}{\sum }\frac{1}{4\pi \varepsilon_{0}}\frac{q_{i}r_{\alpha ,i}}{r_{i}^{3}}$ is the Cartesian component of the electrostatic force, and ε_0_ is the vacuum dielectric constant (the metal ion is assumed to be at the origin of the coordinate frame). Diagonalization of the EFG tensor provides its principal values and PAS relative to the molecular coordinate system, which is expected to be similar to that of the ZFS.

## Results and Discussion

Our experimental approach for determining the ZFS PAS within the Gd­(III) complex structure consists of the following five steps:

(i) Design and synthesize two model complexes, comprising the same Gd­(III) chelate but bearing ^19^F atoms at two distinct positions with respect to Gd­(III), and determine their structures by DFT. In this work, the two Gd­(III) chelates selected are part of the commonly used spin labels, **Gd-DO3A** ^30^ and **Gd-PyMTA** ^34^ (Figure 1, left box).
(ii) Record echo-detected EPR (ED-EPR) spectra between 1.6 and 6 K and simulate the spectra to determine *D*, *E,* and their distributions. This simulation yields the orientations of the ZFS tensor, θ_0_ and φ_0_, relative to the external magnetic field, of the molecules excited at each field position within the EPR spectrum.
(iii) Record ^19^F OS-ENDOR spectra across a wide range of magnetic fields outside the CT. Using the ranges of θ_0_ and φ_0_ contributions for each measurement (determined in step (ii)), the ENDOR spectra are jointly simulated, providing the orientation of the Gd–F vector, γ and ρ, relative to the ZFS PAS.
(iv) Determine the ZFS PAS in the Gd­(III) chelate from the two sets of γ and ρ values for the two model complexes with the same Gd­(III) chelate.
(v) ^1^H OS-ENDOR spectra of the closest surroundings of the Gd­(III) chelate were recorded and analyzed to resolve ambiguities in PAS alignment, arising from the spin Hamiltonian symmetry.

**Figure 1:**
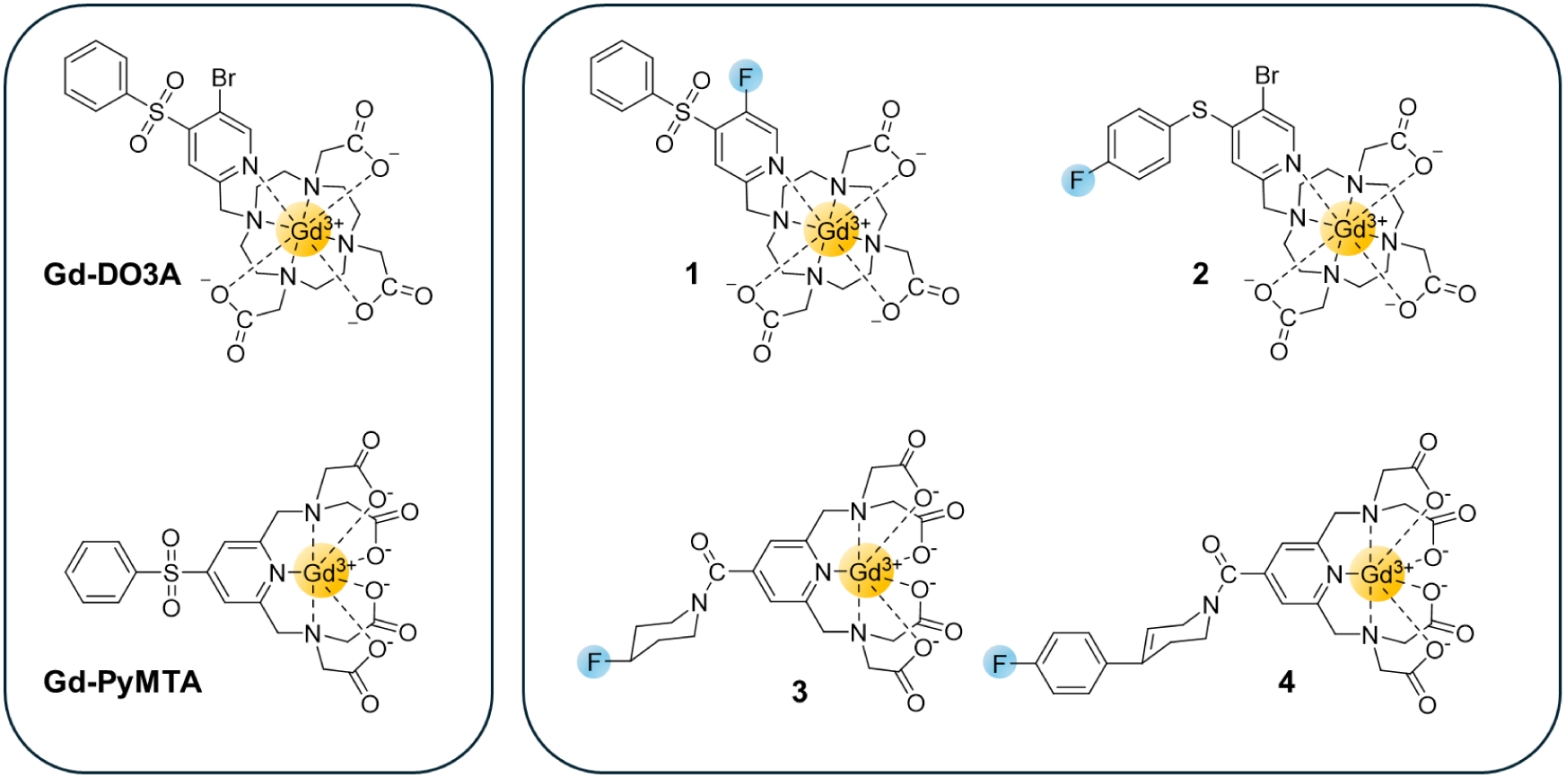
Chemical structures of the free spin labels **Gd-DO3A** and **Gd-PyMTA** (left box) and the fluorinated model complexes used in this work, comprising the same Gd­(III) chelates (**1** and **2** for **Gd-DO3A** and **3** and **4** for **Gd-PyMTA**, right box). **Gd-DO3A** and **Gd-PyMTA** chelates have one and two first-sphere H_2_O ligands, respectively, which are omitted in the schemes for clarity.

The detailed results for each step specified above, are provided next.

### The Model Complexes and Their Structures

The structures of the two pairs of model complexes chosen for this work are given in Figure 1 (right box). Complexes **1** and **2** comprise the chelate of the spin label **Gd-DO3A**, and complexes **3** and **4** constitute the chelate of the **Gd-PyMTA** spin label. In this work, we refer to these chelates as **Gd-DO3A** and **Gd-PyMTA.** The ZFS is determined by the close coordination sphere of the Gd­(III) ion; therefore, it is not expected to be altered by the fluorine moiety attached to the periphery of the complex. The determination of the ZFS PAS in the molecules requires knowledge of their structures, preferably with a single dominant conformer. The optimized structures of the four complexes were calculated by DFT (see Section S2). The potential conformational diversity of the complexes was inspected to identify possible isomers, conformers, and rotamers that may have distinct ENDOR spectra. We found that each of the complexes **1–4** possesses one dominant conformation (see Section S2 and Figures S2–S4).

To display and compare the ZFS PAS orientations for the two types of Gd­(III) chelates (**Gd-DO3A** and **Gd-PyMTA**), we selected the following two fixed, characteristic structural vectors within the geometry of each complex:

(A) the vector connecting the Gd atom with the N atom of the coordinating pyridyl group (for both **Gd-DO3A** and **Gd-PyMTA**);
(B) the normal to the NNNN plane created by the four nitrogen atoms of the cyclen group of **Gd-DO3A**;
(C) the normal to the plane of the pyridyl ring of **Gd-PyMTA**

Each PAS orientation is characterized by the three direction cosines of these, A, B and C, vectors relative to the PAS. Accordingly, the PAS of **Gd-DO3A** is described by direction cosines A­(*a*_x_, *a*_y_, *a*_z_) and B­(*b*_x_, *b*_y_, *b*_z_), and that of **Gd-PyMTA** by A­(*a*_x_, *a*_y_, *a*_z_) and C­(*c*_x_, *c*_y_, *c*_z_), where all *a*_*i*_, *b*_*i*_, and *c*_*i*_ are chosen to be positive.

### ED-EPR of the Model Complexes

The echo-detected EPR (ED-EPR) spectra of **Gd-DO3A** and **Gd-PyMTA** recorded at 3.6–4.2 K are shown in Figure 2A,B, along with their simulations, where the contributions of each of the individual EPR transitions to the simulated spectra are plotted. To increase the stability and confidence level of the fit, five EPR spectra, recorded between 1.6 and 7.5 K, were jointly simulated (see Section S3 and Figure S5). In this temperature range, the W-band EPR spectra significantly change their shapes due to the variations in electron-spin energy level populations. The best-fit distributions of *D* and *E* are listed in Table 1. The ranges of the ZFS orientations, θ_0_ and φ_0_, selected at the four field positions, a–d, within the spectra are presented in Figure 2C,D. They illustrate that orientation selection is significant and that different ranges of θ_0_ and φ_0_ are selected at each of the field positions. The ED-EPR spectra for the models with the same chelate have the same line shapes; therefore, the ZFS parameters are not affected by the remote moiety consisting of the ^19^F nucleus.

**Figure 2:**
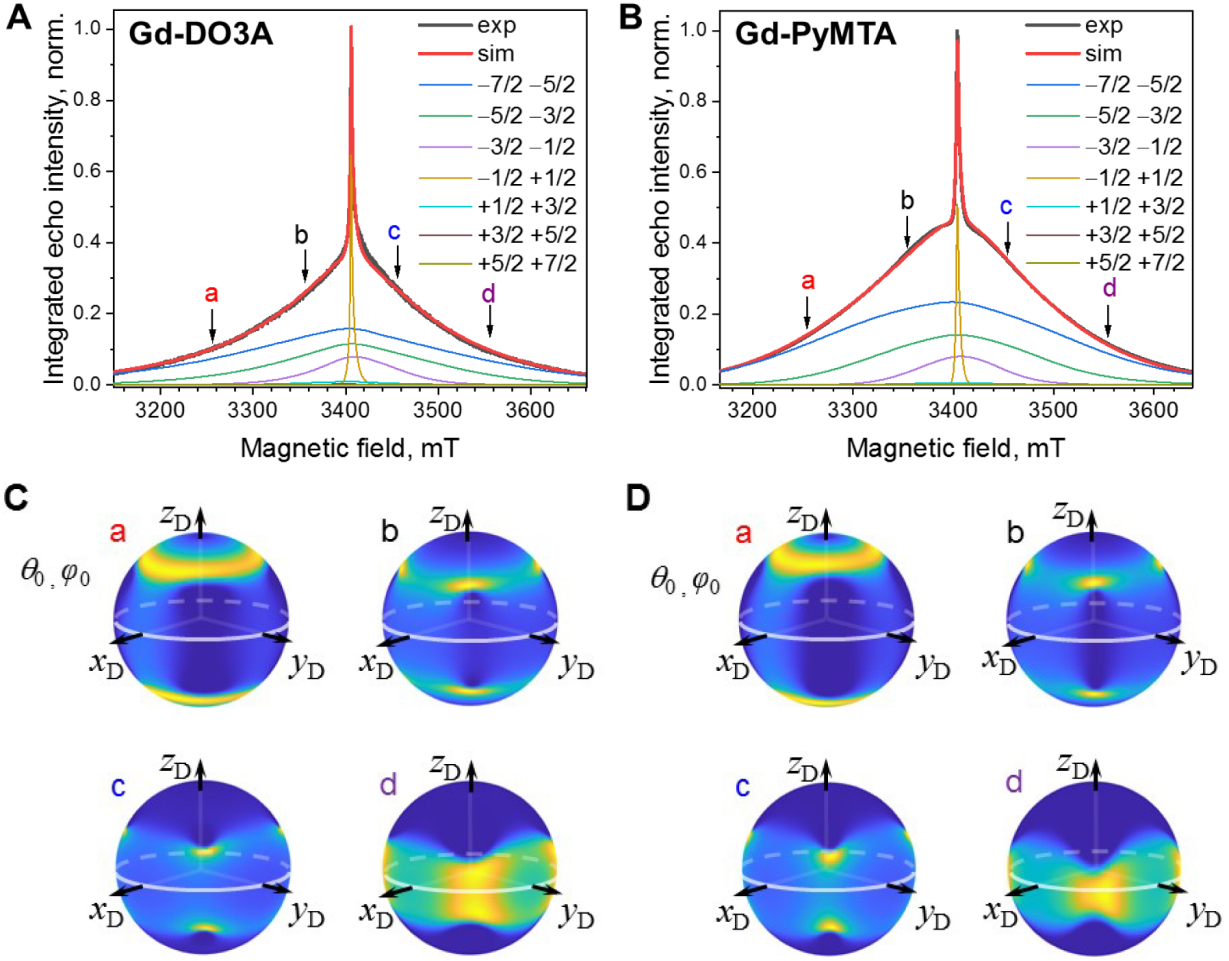
(**A, B**) W-band ED-EPR spectra of **Gd-DO3A** (in labeled **Ub-T66C**) recorded at 4.2 K (A) and **Gd-PyMTA (**complex **3**) recorded at 3.6 K (B). Experimental ED-EPR spectra are in black, simulations are in red, and the contributions of individual electron spin transitions are shown in different colors. (**C, D**) Heat plots of the contributions of different molecular orientations (shown as the magnetic field orientation in the ZFS molecular reference frame) to the spectra recorded at field positions a–d of **Gd-DO3A (C)** and **Gd-PyMTA (D)** chelates, respectively.

**Table 1:** Parameters of the Gaussian Distributions for *D* and *E* Derived from Simulations of the ED-EPR Spectra: Mean Values (*D*_0_ and *E*_0_) and Gaussian Distribution Widths (Δ*D* and Δ*E*), as Defined by (Eq. S1)­^a^

Complex	*D*_0_, MHz	Δ*D*, MHz	*E*_0_, MHz	Δ*E,* MHz
**Gd-DO3A**	–1190 ± 50	1570 ± 50	–95 ± 25	810 ± 50
**Gd-PyMTA**	–960 ± 50	950 ± 50	–80 ± 20	820 ± 50

a Parameters uncertainties were obtained from the covariance matrix of the nonlinear least-squares fit.

### ^19^F OS-ENDOR of **Gd-DO3A**

The ^19^F OS-ENDOR spectra of **1** recorded at 4 K are presented in Figure 3A. At this temperature, the −7/2 ↔ −5/2, −5/2 ↔ −3/2, and −3/2 ↔ −1/2 transitions are appreciably populated. Accordingly, one can readily identify the perpendicular ENDOR singularities corresponding to *m*_S_ = −7/2, −5/2, −3/2, and −1/2. These features appear to the left of the ^19^F Larmor frequency, ν_I_, and are separated by *a* = 370 kHz, consistent with the hyperfine coupling constant *a*_⊥_, determined from the CT ENDOR spectrum (top spectrum in Figure 3A). The corresponding parallel-orientation singularities for the same *m*_S_ values appear to the right of ν_I_ and are most clearly observed in the spectra recorded with shorter τ values (Figure S6).^31^ Figure 3A shows pronounced orientation selection, reflected primarily in the relative intensities of the parallel singularities, which markedly increase at higher fields. Joint simulation of the full set of spectra in Figure 3A and S6 (Section S4) yielded hyperfine parameters, listed in Table 2, and provided γ_1_ = 64 ± 9° and ρ_1_ = 74 ± 28° that define the orientation of the Gd–F dipolar axis in the ZFS PAS.

**Figure 3:**
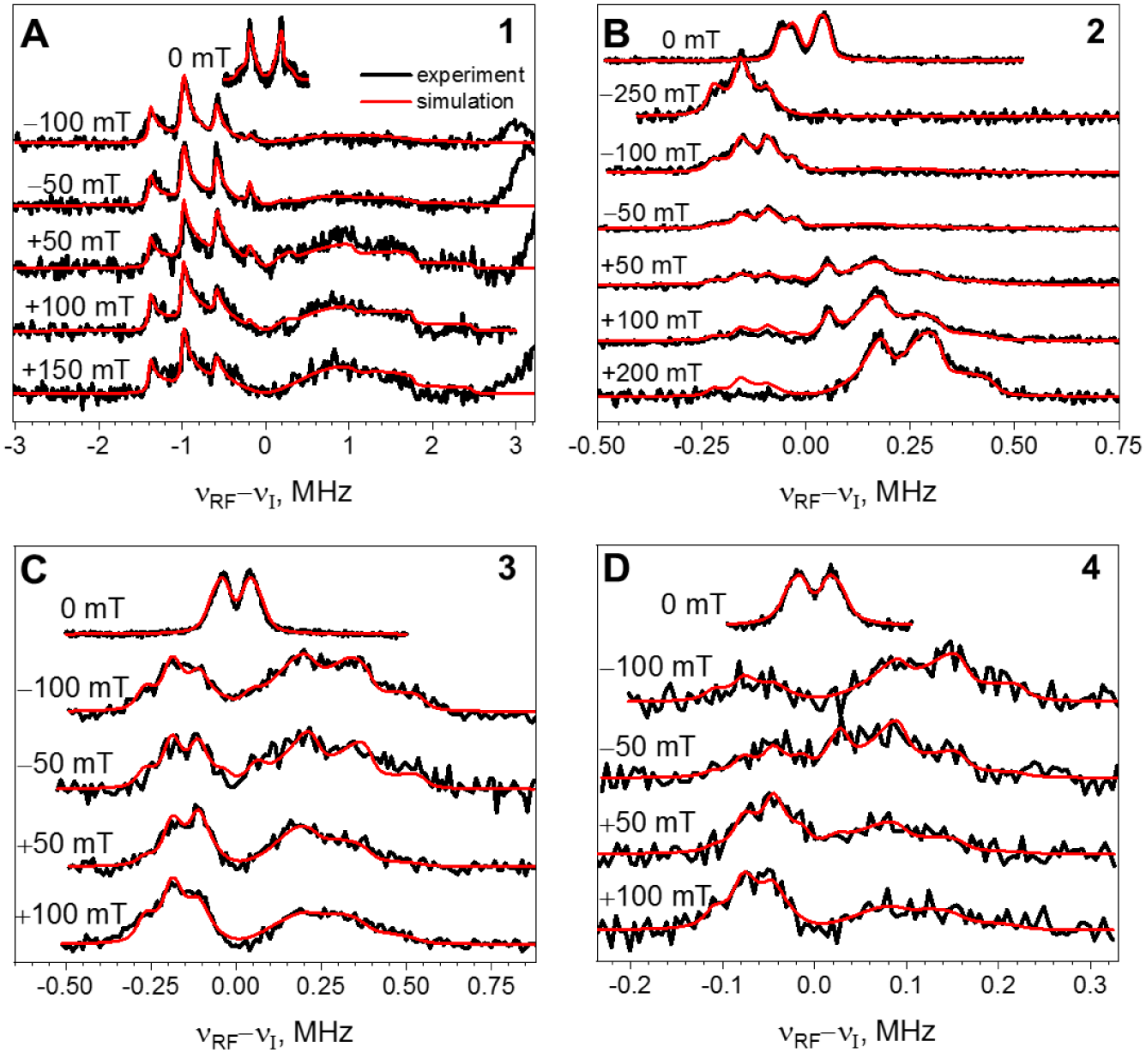
W-band Mims ^19^F OS-ENDOR spectra of complexes **1–4** (**A**–**D**). The spectra were recorded at 4 K with τ = 1 μs (for **1**) and τ = 2 μs (for **2**–**4**). The magnetic field shift from the CT is listed for each spectrum. The red lines are the simulations obtained with the parameters given in Table 2. The contributions of individual electron spin manifolds are shown in Figure S8.

**Table 2:** List of the Parameters Obtained from the Simulations of the ^19^F OS-ENDOR Spectra of the Model Complexes^a^

Sample	*a*_⊥_, kHz	*r,* Å	*a*_iso_, kHz	γ, deg	ρ, deg
**1**	369 ± 1	5.85	–28.0 ± 1.6	64 ± 9	74 ± 28
**2**	65.1 ± 0.1	10.5	0	90 ± 5	72 ± 7
**3**	78.9 ± 0.3	9.8	0	48 ± 4	62 ± 17
**4**	32.5 ± 0.4	13.2	0	45 ± 8	0 ± 38
**GB1-Q32C Gd-DO3A**	19.4 ± 0.3	15.6	0	90 ± 13	84 ± 18
**Ub-M1C Gd-DO3A**	67.5 ± 0.1	10.3	0	40 ± 3	46 ± 17

a Uncertainties of the hyperfine constants were obtained from the covariance matrix of the nonlinear least-squares fit, while uncertainties of the angular parameters were determined by parameter shifting until the increase in the objective function exceeded the noise level (see Section S4).

To obtain sufficient information for determining the ZFS PAS within the molecule, we additionally investigated model complex **2**. It possesses the same **Gd-DO3A** core but with an ^19^F atom at a different position. The ^19^F OS-ENDOR spectra of **2**, presented in Figure 3B and S7, display a distinctly different behavior from **1**; the perpendicular singularities diminish much more strongly and are nearly absent at +200 mT. Concomitantly, the intensities of the parallel singularities increase with the field. The spectra were simulated with the parameters listed in Table 2. The pronounced differences between the spectra recorded at ± 200 mT corroborate the strong orientation selection across the noncentral Gd­(III) EPR transitions (Figure 2C). The orientation of the Gd–F vector in **2** is described by polar angles γ_2_ = 90 ± 5° and ρ_2_ = 72 ± 7°.

### Determination of the ZFS Orientation

With the orientations of the two Gd–F vectors, given by γ_1,2_ and ρ_1,2_, in complexes **1** and **2**, sharing the same Gd­(III) chelate, we proceeded to determine the ZFS PAS. First, the DFT-optimized geometries of **1** and **2** were aligned so that the atoms in the **Gd-DO3A** chelate overlap, producing a combined structural model in which the Gd­(III) ion is placed at the origin. We then searched for the orientation of the ZFS PAS in this combined model that reproduces the experimentally determined γ_1,2_ and ρ_1,2_ values. Details of the structural alignment procedure are provided in Section S5. In principle, the orientations of two independent vectors uniquely define a coordinate system, and this information should be sufficient to determine the ZFS PAS. However, due to the orthorhombic symmetry of the ZFS spin Hamiltonian (eq 1),^19^F OS-ENDOR cannot distinguish fluorine positions that are symmetric with respect to the ZFS PAS coordinate planes. Consequently, two inequivalent orientations of ZFS PAS satisfy γ_1,2_ and ρ_1,2_ equally well (details in Section S5, Figure S9). A conceptually related analysis was reported for Mn­(II) in a superoxide dismutase variant, where orientation-selective ^1^H and ^14^N ENDOR were used to infer the local ZFS tensor.^35^

For both possible ZFS PAS obtained for **Gd-DO3A,** the *y*_D_ axis is approximately along the Gd–N­(pyridyl) bond (vector A). However, the directions of *x*_D_ and *z*_D_ axes are swapped between the two variants (Figure S10A,B): in the first, the normal to the NNNN plane of the cyclen fragment (vector B) aligns with *z*_D_, while in the second, it aligns with *x*_D_. Only one of these can correspond to the true ZFS PAS orientation. To resolve this ambiguity, we turned to ^1^H OS-ENDOR.

### ^1^H ENDOR

First, we recorded ^1^H spectra at the CT. The good agreement between the experimental ^1^H ENDOR spectra and simulations based on the DFT-optimized Gd­(III)-chelate structure (Figure S11A and Table S1) corroborates the predicted Gd–H distances. This agreement suggests that simulations of ^1^H OS-ENDOR using the predicted hydrogen atom positions should be capable of reproducing the orientation-selection behavior.

The experimental ^1^H OS-ENDOR spectra of the free **Gd-DO3A** label recorded at magnetic fields ± 200 mT from the CT are shown in Figure 4A. To enhance the signals of the protons located closest to the Gd­(III) ion, relatively short τ values (120–350 ns) were employed. Because 26 hydrogen atoms surround the Gd­(III) ion, each with a different orientation relative to the ZFS axes, their individual orientation-selection contributions largely cancel, resulting in a weak net effect. Nevertheless, a weak but distinct orientation-selection signature is observed experimentally, with the contribution from parallel orientations (ν_RF_ > ν_I_) increasing at lower fields.

**Figure 4:**
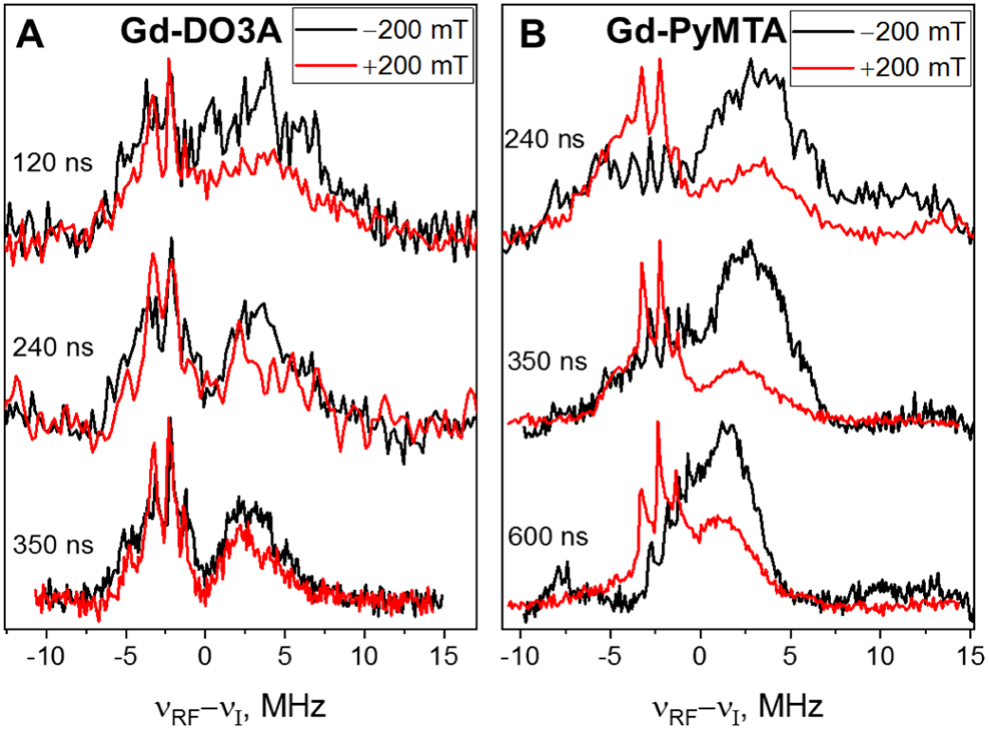
^1^H OS-ENDOR spectra of the **Gd-DO3A** spin label (**A**) and complex **3** (**B**) in a fully deuterated solvent (D_2_O:glycerol-d_8_ 3:1), recorded at field positions −200 mT (black line) and +200 mT (red line) relative to the CT. Three different values of τ (120 ns, 240 ns, 350 ns and 240 ns, 350 ns, and 600 ns) in the Mims ENDOR sequence were used for the spectra in (**A**) and (**B**), respectively.

The DFT-optimized hydrogen positions were used to predict the ^1^H OS-ENDOR spectra for the two possible PAS orientations without any fitting parameters (Figure S10C,D). For the structure displayed in Figure S10A, the experimental orientation selection pattern is reproduced, while for the second variant, no orientation selection is predicted. We therefore conclude, based on the experimental ^19^F and ^1^H OS-ENDOR data, that the orientation of the ZFS PAS corresponds to the structure shown in Figure S10A. This orientation is shown in Figure 5A,B.

**Figure 5:**
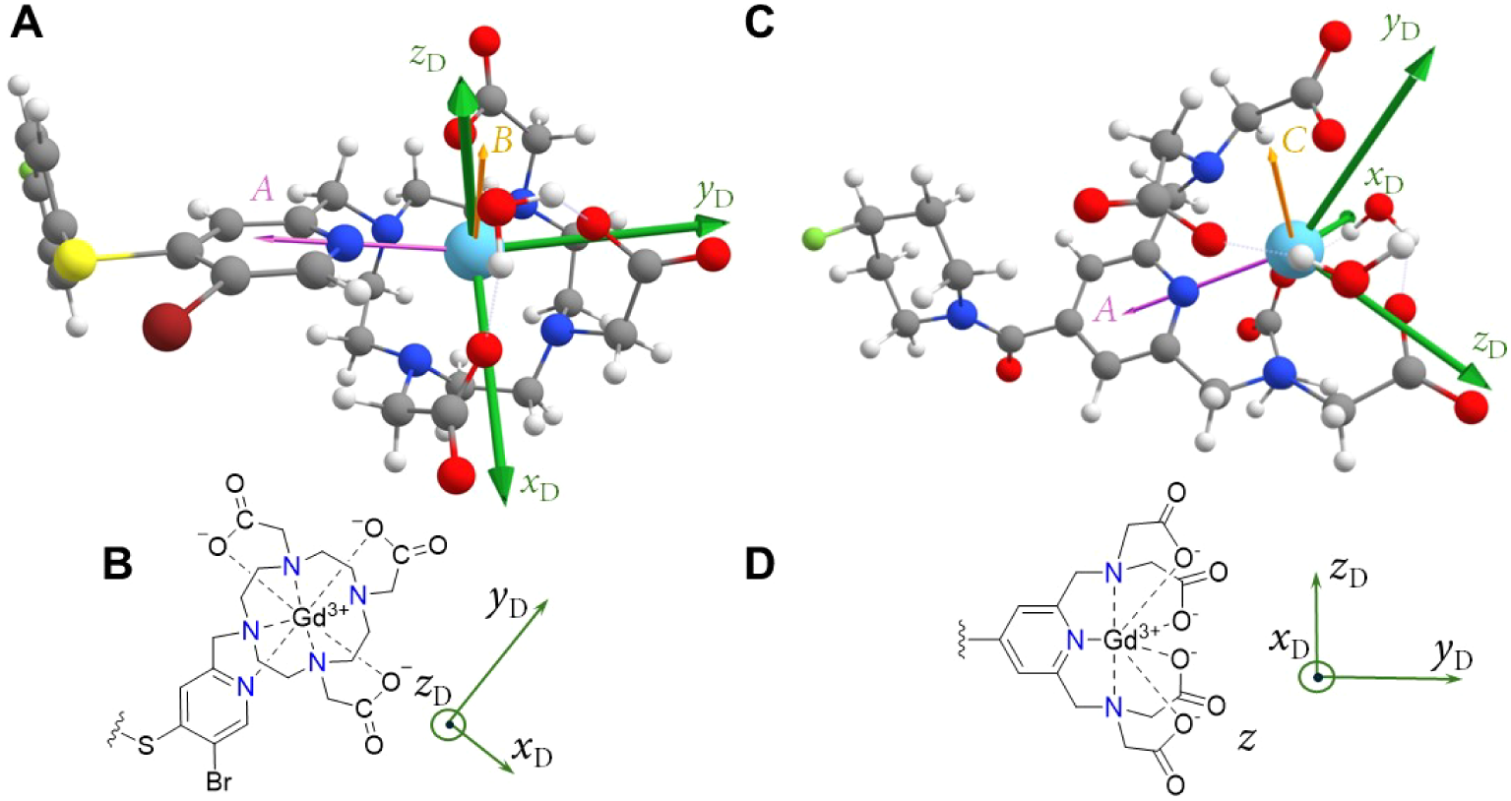
**(A, C)** Optimized structures of complexes **2** (**A**) and **3** (**C**), and the location of the ZFS PAS directions in these structures. Carbon atoms are shown as gray, nitrogen as blue, sulfur as yellow, oxygen as red, bromine as brown, fluorine as green, gadolinium as cyan, and hydrogens as white spheres. Magenta arrows show molecular vectors A, and yellow arrows show molecular vectors B (for the **Gd-DO3A** chelate) and C (for the **Gd-PyMTA** chelate), correspondingly. (**B, D**) Simplified chemical structures of the **Gd-DO3A** (**B**) and **Gd-PyMTA** (**D**) chelates, with the positions of ZFS PAS of Gd shown schematically with green arrows. In (**B**), the H_2_O ligand is coordinated along the *z*_D_ axis, and in (**D**), the two H_2_O ligands are coordinated along the *x*_D_ axis, on both sides of the drawing plane.

### ^19^F OS-ENDOR **Gd-PyMTA**

The same sequence of experiments and analyses was carried out for **Gd-PyMTA**. The ^19^F OS-ENDOR spectra of **3**, shown in Figure 3C, displayed only minimal orientation selection, whereas **4** revealed that the parallel singularities dominate at the negative magnetic field shifts, while the perpendicular singularities become more dominant at positive magnetic field shifts, a behavior opposite to what was observed for **Gd-DO3A** (Figure 3D). Simulation of the spectra yielded the γ_1,2_ and ρ_1,2_ angles listed in Table 2. As in the case of **Gd-DO3A**, these angles correspond to two possible orientations of the ZFS PAS (shown in Figure S12A,B) that cannot be distinguished by ^19^F ENDOR alone, requiring ^1^H ENDOR spectra for identifying the correct one.

Strong orientation selection is observed in the ^1^H ENDOR spectra of **Gd-PyMTA** (Figure 4B), most likely because only 12 hydrogens are in the immediate coordination sphere of Gd­(III), featuring a more anisotropic spatial distribution than that for **Gd-DO3A**. Clearly, the ^1^H ENDOR data are only compatible with the structure displayed in Figure 5C,D and S12A. In this structure, the *y*_D_ axis is the one closest to the bond between Gd and the pyridyl nitrogen, whereas the *x*_D_ axis is close to perpendicular to the pyridyl group.

### AIMD Calculations

After the ZFS PASs in **Gd-DO3A** and **Gd-PyMTA** were experimentally determined, two questions still need answers: (1) ENDOR spectra simulations showed that the directions of ZFS principal axes are relatively well-defined and therefore may be represented by a single set of angles with relatively small uncertainties, despite the broad distribution of the *D* and *E* values. Can this result be theoretically reproduced and the spread of the principal axes quantified? (2) How well can the orientation of the principal axes of ZFS in the molecule be predicted theoretically?

Although considerable attention has been devoted to *ab initio* prediction of the ZFS tensor of Gd­(III) chelates,^12,21,22,36,37^ these calculations cannot be considered routine. Because the ZFS of Gd is very small on the energy scale, the current techniques struggle to reproduce even the sign and order of magnitude of *D* and *E*.^38–40^ Superposition approaches have proven to be useful for the estimation of the ZFS symmetry and distributions.^20,33,40^ These approaches treat the total ZFS on the metal center as a superposition of contributions from different ligands. Here, we use such a method to estimate the distribution of the ZFS orientations based on the conformational ensemble of the complex. To this end, we performed *ab initio* molecular dynamics (AIMD) simulations of the studied systems. For computational efficiency, Gd­(III) was replaced with Y­(III). This produced very similar geometries because the ionic radii of the two ions are close (Figure S13). MD trajectories were generated for several starting geometries of the **Y-DO3A** and **Y-PyMTA** chelates to sample the conformational space present in solution (see the Experimental Section for more details). At each MD step, the chelate geometry and Mulliken charges were used to compute the EFG tensor on the metal ion employing eq 11, which was used as a proxy for the ZFS tensor. Diagonalization of this tensor yielded its principal values and the PAS.

The results obtained for **Y-DO3A** are shown in Figure 6A. The principal values of EFG were normalized to the median *V*_zz_ value, V̅_zz_ (first panel in Figure 6A), allowing comparison to the experimentally obtained distributions of ZFS principal values (*D*_xx_, *D*_yy_, and *D*_zz_), also normalized to the median value of *D*_zz_, D̅_zz_ (second panel in Figure 6A). The two lower panels show the distributions of direction cosines A (*a*_x_, *a*_y_, *a*_z_) and B (*b*_x_, *b*_y_, *b*_z_), which define the direction of the bond between the metal ion and pyridyl nitrogen (A) and the normal vector to the NNNN plane of the cyclen ring (B). The vector B is also close to the coordination direction of the H_2_O ligand in the inner shell of Gd­(III). The experimentally determined direction cosines are shown as solid vertical lines in the same panels. These can be compared with the mean orientations of A and B taken over all AIMD trajectories (shown as dashed vertical lines).

**Figure 6:**
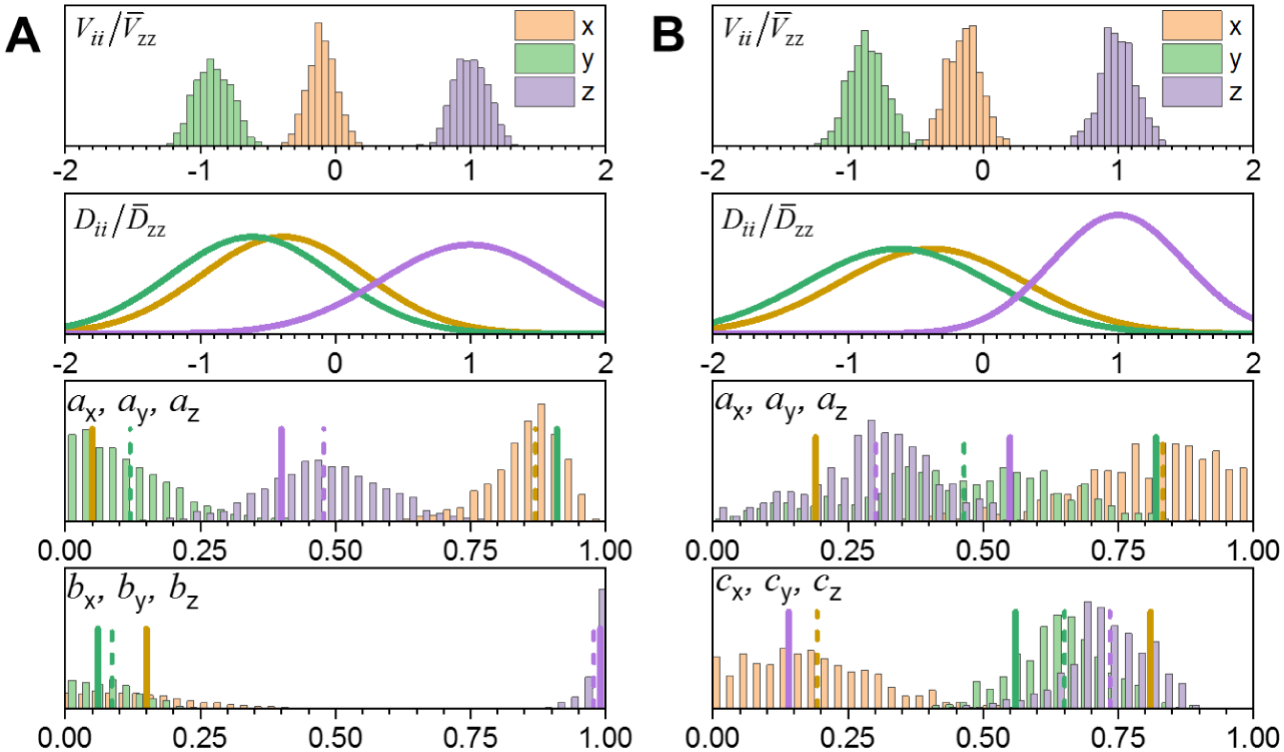
Histograms and graphs depicting the distributions of normalized principal values of the EFG tensor obtained from AIMD simulations (first panel) and normalized experimentally determined principal values of the ZFS tensor (second panel), and the direction cosines defining the PAS of the EFG tensor obtained from AIMD simulations (two lower histograms). In the two lower panels, the experimental ZFS PAS obtained from ENDOR data is shown as vertical solid lines, and the mean orientation of EFG PAS over all AIMD trajectories is shown as vertical dashed lines. The data are shown for **Gd/Y-DO3A** (**A**) and **Gd/Y-PyMTA** (B) chelates.

For **Gd-DO3A**, the experimentally determined PAS closely matches the calculated one, aside from a swap of the *x*_D_ and *y*_D_ principal axes. In both cases, the *z*_D_ axis is oriented strictly normal to the cyclen plane. This indicates that the cyclen moiety is the primary structural element defining both electrostatic and magnetic anisotropy, with the pyridyl substituent acting as a secondary perturbation. The predicted PAS distribution is relatively narrow, and the orientations agree reasonably well with the experimental results; however, the predicted rhombicity is clearly different. Interestingly, a previously published CASSCF calculation performed on the Gd-HPDO3A complex^22^ predicted *z*_D_ to lie within the cyclen plane rather than along its normal.

For **Y-PyMTA** (see Figure 6B), a similar procedure was carried out. Here, the vector A also correspond to the bond between the metal ion and pyridyl nitrogen, while the C vector describe the normal to the pyridyl ring. For **Y-PyMTA,** the predicted PAS distribution is broader than that for **Y-DO3A**, particularly for the A direction. The mean values of the direction cosine distributions agree with the experiment, but the axes order does not: the experimental axis closest to the Gd–N­(pyridyl) direction is *y*_D_, whereas the EFG calculation assigns it as *x*_D_. As for **Y-DO3A**, the predicted tensor exhibits a larger rhombicity and a smaller distribution of principal values than those experimentally observed.

In summary, approximating the ZFS PAS by the EFG PAS provides a reasonable prediction of the ZFS orientation. However, because ZFS depends nonlinearly on EFG, the magnitudes of tensor components, including their rhombicity, and the ordering of the principal axes are not reproduced. A higher level of theory is, therefore, required for quantitative predictions.

Finally, we return to the question raised at the beginning of this section concerning the magnitude of the EFG PAS fluctuations relative to the molecular geometry. These fluctuations can be characterized by the mean-squared angular deviation of vectors A and B in **Y-DO3A** and vectors A and C in **Y-PyMTA**, from their respective average orientations. This gave 7° for A and 9° for B in **Y-DO3A** and 10° for A and 14° for C in **Y-PyMTA,** indicating that in both chelates, the EFG anisotropy axes are sufficiently well-defined within the molecular frame. Although the relationship between EFG and ZFS tensors is not a direct one, the narrow distribution of the EFG axes makes it reasonable to expect that the axes of ZFS are similarly well-defined. This expectation is experimentally supported by the strong orientation selection observed in ^19^F and ^1^H ENDOR spectra of the studied chelates.

For the sake of completeness, we have also estimated the ZFS tensor orientation of the chelates at their optimal geometry using an *ab initio* method (see Section S8 for more details). We found (Figure S14) that the principal axis orientations are consistent with those obtained from EFG tensors, except for some rotation of *y*_D_ and *z*_D_ axes in **Gd/Y-PyMTA**. This reiterates the conclusion that the ZFS principal axis directions are well reproduced by theoretical methods, but the axis indices may get swapped. This is in contrast to the values of *D* and *E*, which differ significantly from of the experimental values. Importantly, it was not possible to carry out the AIMD calculations using the *ab initio* method because of problems with CASSCF convergence.

### Elucidating Protein Structural Features Using ^19^F OS-ENDOR

After determining the ZFS PAS in the structure of Gd­(III) spin labels, we next applied ^19^F OS-ENDOR to fluorine-labeled proteins for extracting the relative position of the ^19^F nucleus with respect to the Gd­(III) spin label. The proteins were **GB1-Q32C Gd-DO3A** with 5-fluorotryptophan (5F-Trp) at position 43 and **Ub-M1C Gd-DO3A** with 4-trifluoromethylphenylalanine (4tFmPhe) at position 45. The CT regions of the ED-EPR spectra of the two labeled proteins and complex **2** are compared in Figure S15. The spectra coincide within the experimental uncertainty; therefore, we conclude that any possible interaction of the label with the protein did not change the ZFS magnitude. Nevertheless, we cannot rule out cases in which the binding to the protein will distort the close coordination shells of the label. Moreover, this may depend on the label itself, with a cyclen-based label such as **Gd-DO3A** expected to be more robust than a label such as **Gd-PyMTA**.

The ^19^F OS-ENDOR spectra for both proteins are presented in Figure 7A,B, showing opposite orientation-selection behavior. In **GB1**, the perpendicular features dominate at −150 mT and the parallel ones at +150 mT, whereas in **Ub,** the parallel features are strongest at lower fields. For **GB1**, the extracted γ and ρ angles (90° and 84°, respectively) indicate that the Gd–F vector is approximately parallel to the ZFS *y*_D_ axis, i.e., aligned with the Gd–N (pyridine) direction (Table 2). In **Ub,** the Gd–F vector is tilted from the normal of the NNNN plane by 40°, with its projection onto the NNNN plane resulting in an angle of 46° from the Gd–N (pyridine) bond (Figure 7C,D). Thus, the opposite orientation-selection behavior reflects the different orientations of the Gd–F vector within the protein structure.

**Figure 7:**
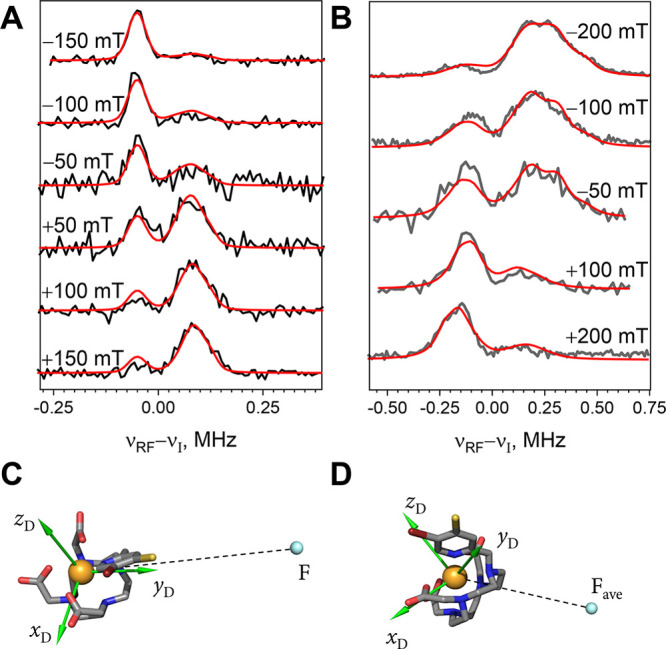
^19^F OS-ENDOR of **GB1-Q32C Gd-DO3A** (**A**) and **Ub-M1C Gd-DO3A (B)** measured at 2.5 K and τ = 4 μs (**A**) and 3.8 K and τ = 2 μs (**B**). The red lines correspond to simulations with the parameters listed in Table 2. (**C, D**) The optimized structures of the label in ZFS PAS with the fluorine atom position obtained from the simulations of ^19^F OS-ENDOR spectra shown in panels (**A**) and (**B**), respectively. For Ub-M1C, F_ave_ represents the average position of the three fluorine atoms in the trifluoromethyl group.

In Figure 7C,D, the structure of the protein is not included. To derive protein structural information from the experimental results, either the known Gd­(III) position can be used for locating the ^19^F position or vice versa. First, we will focus on determining the position of Gd­(III) relative to the known ^19^F location. Although the Gd­(III) spin label itself is rigid, rotations around the tether C–S bond and other bonds within the cysteine amino acid are possible, leading to multiple Gd­(III) positions. When the protein structure is known, these positions can be determined *in silico* using established software packages such as MMM,^41^ MtsslSuite,^42^ and chiLife.^43^ For both proteins, *in silico* labeling with MtsslSuite predicts a broad distribution of Gd­(III) positions and Gd–F vector orientations (Figure S16A-C). Using these distributions to simulate the expected ^19^F OS-ENDOR spectra for **GB1-Q32C** (Figure S16D) resulted in poor agreement with the experiment. Although the predicted splitting is correct, the orientation selection pattern is not reproduced. This indicates that the Gd­(III) labeling site has significantly less conformational freedom than predicted by the modeling. Similar calculations were done with Gd­(III) locations predicted by MMM, and the agreement with the experimental ENDOR spectra was not satisfactory (Figure S17). The quantitative estimation of the accessible conformational space, presented in Table S2, demonstrates that inclusion of the ENDOR-derived angular constraints markedly reduces the number of allowed spin-label conformers for both studied proteins. For example, out of 201 conformations predicted by MtsslSuite for **GB1-Q32C**, most (184) are consistent with the experimental Gd–F distance. However, only 45 out of them are also consistent with the experimental angles γ and ρ (see Table S2 and accompanying text for more details).

Conversely, the experimentally determined *r*, γ, and ρ values can be used to position the Gd­(III) ion within the structure of the protein. This requires two additional geometric constraints: (i) The sulfur atom of the attachment cysteine must superimpose on the covalently attached sulfur atom of the label (a distance threshold of 2 Å was chosen to screen possible label positions), and (ii) no steric clashes are allowed between the protein and the spin label (see Section S10 and Figure S18 for details). Applying this procedure narrows the distribution of allowed spin label positions markedly (Figure S16A,B). Figure 8 shows a representative structure for each protein and the full sets of structures compatible with the OS-ENDOR experiment. The imposed geometric constraints are provided in Figures S19 and S20.

**Figure 8:**
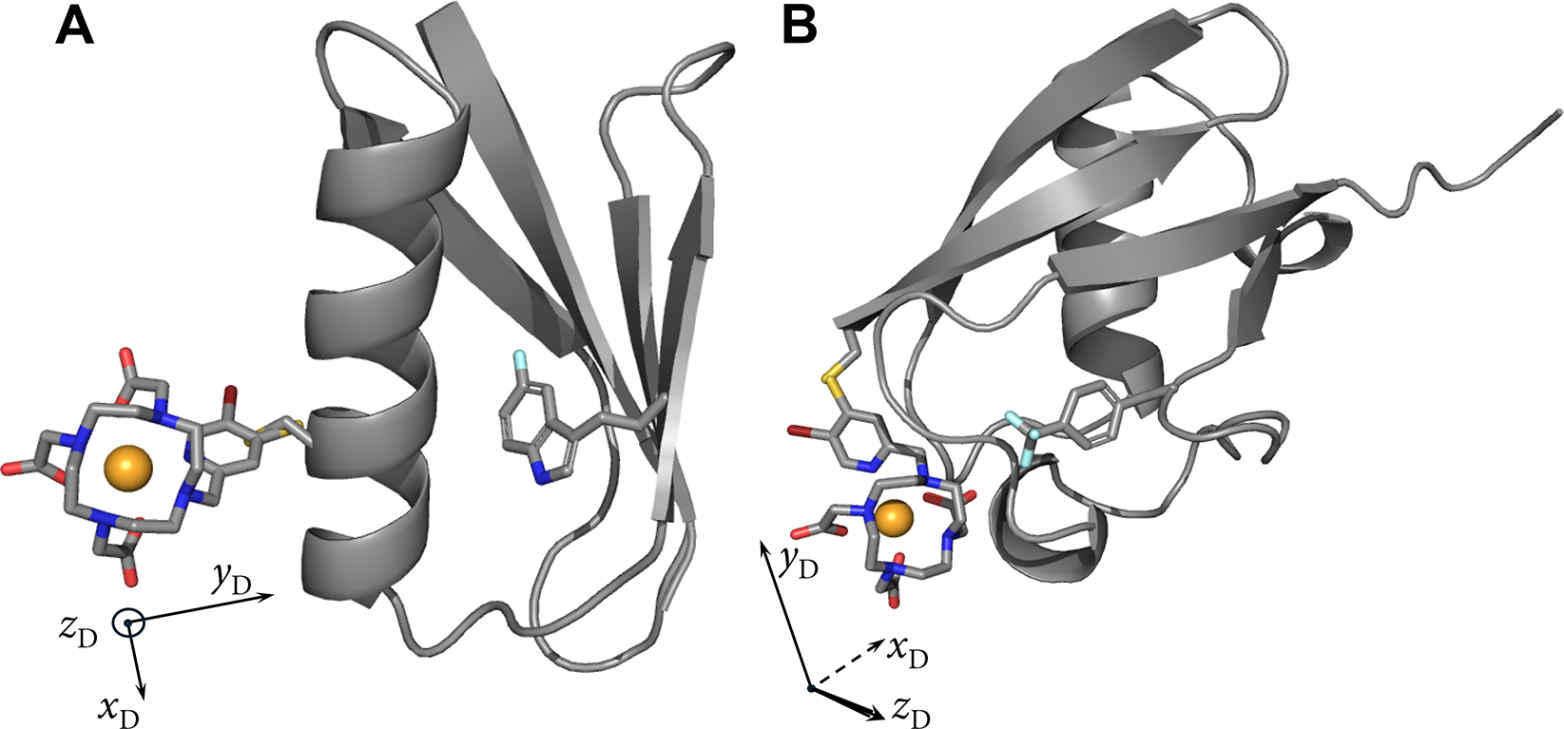
Structures of **GB1-Q32C** (**A**) and **Ub-M1C** (**B**) shown in ribbon representation (PDB IDs 1GB1 ^44^ and 1UBQ,^45^ respectively) with one of the spin label positioned using ^19^F OS-ENDOR data, as detailed in Section S10. The ZFS PAS of Gd­(III) is indicated by the coordinate system in the figure. The full set of all obtained structures is shown in Figures S19 and S20.

The label positions, which are consistent with the ^19^F OS-ENDOR data, occupy only a small subset of the conformational space predicted *in silico* (Figure S16A, B). Interestingly, in the case of **Ub-M1C**, a mismatch between the *in silico* distance distributions and both ^19^F PRE and ENDOR experiments was noted previously.^24,46^ Thus, the ^19^F OS-ENDOR-refined label positions suggest that the spin label engages in specific interactions with the protein, preferentially occupying the position adjacent to the **Ub** loops. Previously, a reduction of conformational space compared to the *in silico* predictions was confirmed for nitroxide-labeled azurin using pulsed double electron–electron resonance and X-ray diffraction, underlining the importance of specific interactions of the label with the protein residues and solvent.^47,48^ Our example shows that the OS-ENDOR data are not limited to filtering predicted Gd­(III) positions based on the available structure, but can give locations not predicted by the rotamer libraries, as in the case of **Ub-M1C**, where the structural changes are imposed by the labeling at the C-terminal.

In our calculation, we treated the ^19^F-labeled residues as fixed within the protein structure. Although tryptophan side chains are not expected to flip in solution and the orientation of 5F-Trp in **GB1** has previously been established by NMR,^49^ we examined a hypothetical scenario in which the indole ring was inverted. In contrast to 4tFmPhe in **Ub**, for which a 180° flip leaves the para-CF_3_ position unchanged, an indole inversion in **GB1** would alter the relative Gd–F geometry (Figure S21). Notably, such an inversion would be expected to shift the Gd–F distance distribution toward longer distances, inconsistent with the experimental ^19^F ENDOR data. For completeness, we nevertheless recalculated the Gd­(III) label positions based on the experimental ^19^F OS-ENDOR spectra, assuming the flipped indole orientation. None of the resulting structures satisfied the imposed geometrical constraints: the sulfur atoms of the label and the cysteine were separated by more than 5 Å, and several atoms in the Gd­(III) label sterically clashed with the protein backbone (Figure S21A-D). These results indicate that the ^19^F OS-ENDOR-derived constraints are sufficient to discriminate between indole orientations and, in principle, they can establish the correct 5F-Trp conformation (or other residues) when not known *a priori* or ruled out solely on the basis of distance measurements. It has recently been demonstrated by Judd et al.^50^ that by labeling the residue at multiple positions with Gd­(III), Gd–F ENDOR distance distributions can be used to determine fluorinated side-chain conformations.

To summarize, orientation selection, combined with the known ZFS PAS orientation of the Gd­(III) label, can be used to determine the Gd­(III) position when the fluorinated moiety is fixed in the protein structure and discriminate between multiple possible conformations of the ^19^F-labeled amino acid.

## Conclusions

A general experimental strategy (Scheme 1) for determining the molecular frame orientation of the tensor in Gd­(III) chelates in frozen solution was developed. The approach combines stepwise simulation and analysis of EPR and ^19^F/^1^H orientation-selective ENDOR spectra with molecular geometry optimization and structural alignment. When applied to the spin labels **Gd-DO3A** and **Gd-PyMTA**, this method yields ZFS PAS relative to the structures, with its orientation remaining well-defined despite substantial *D* and *E* distributions.

**Scheme 1:**
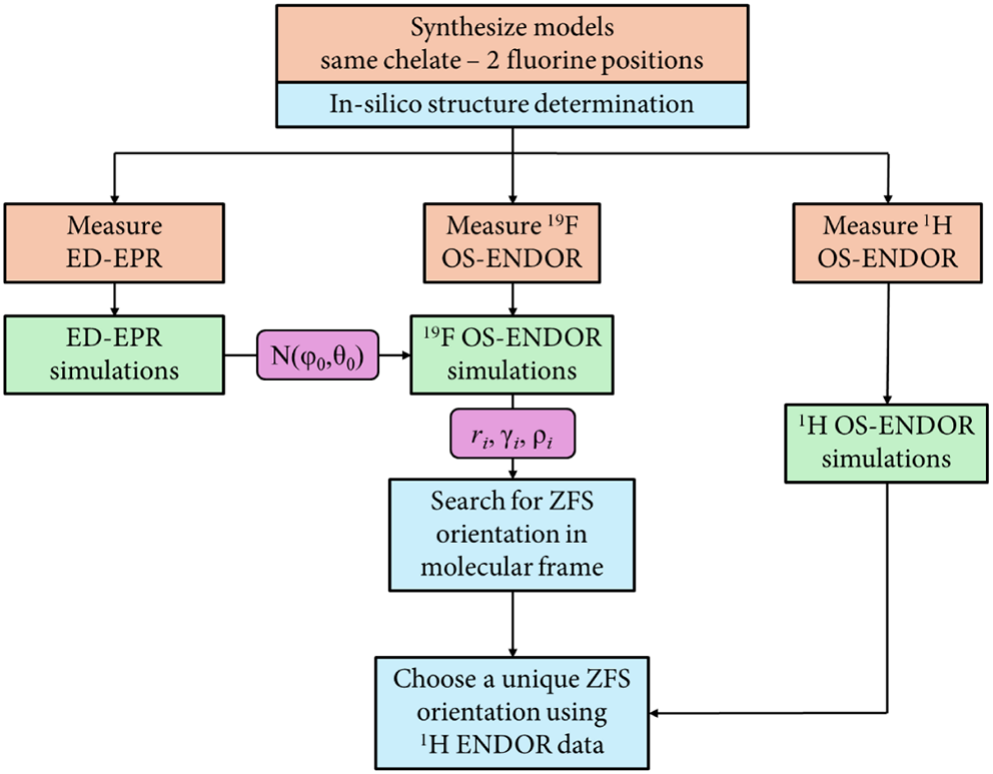
Summary of the General Approach for Gd­(III) ZFS Orientation Determination from OS-ENDOR Measurements^a^

a Orange-colored boxes correspond to experiments, green boxes to spectral simulations, blue boxes to molecular modeling, and magenta boxes to parameters that are passed on to different steps in the procedure.

Access to the ZFS anisotropy enables two modes of Gd­(III) spin label applications. In the first mode, Gd–F distances are measured directly either from the ^19^F ENDOR spectra recorded at the CT or, when these spectra are unresolved, from the splitting between the parallel and perpendicular singularities in off-CT ENDOR spectra. In the second mode, full three-dimensional positioning of the spin label is achieved using OS-ENDOR on noncentral transitions. Demonstrations of this approach for two **Gd-DO3A**/^19^F-labeled proteins permitted positioning of the spin label within each protein and revealed that commonly used rotamer search approaches overestimated conformational disorder. The experimentally derived constraints, therefore, provide a means to refine rotamer searches in spin labeling. Moreover, the presented method is shown to be beneficial for identifying situations in which the spin label adopts an unusual conformation due to the interaction of the label with the protein surface, causing a strong deviation between the predicted conformation space of the label and the ENDOR experiment, like in the example of **Ub-M1C** described above.

The possibility of turning the orientation selection effects on and off by measuring the spectra of different electron spin manifolds is reminiscent of recent developments in Cu­(II) spin labeling, where the presence of orientation selection can be controlled via the choice of Cu­(II)-complexing ligands.^51^

Although the orientation selection effects are well-pronounced in ENDOR experiments, as described here, they have not been systematically studied in pulsed dipolar electron–electron resonance (DEER) spectroscopy for Gd­(III). The field dependence of DEER traces was only reported in the context of evaluating the effects of the pseudosecular spin–spin interactions for short-distance Gd–Gd dimers.^52,53^ For Gd-nitroxide DEER, orientation selection effects due to the nitroxide anisotropy were reported and analyzed.^54^ In RIDME experiments, performed on the CT, any orientation selection effects are unlikely; however, they might in principle be observed in DEER experiments, especially if excitation pulses are applied farther away from the CT, where the orientation selection is expected to be maximal (cf. Figure 2C,D). Our results call for further systematic studies of OS-DEER for the possibility of extracting additional structural information from Gd­(III) spin labels.

The proposed methodology for ZFS determination is broadly applicable: it can be extended to nuclei other than ^19^F and to other half-integer high-spin ions such as Mn­(II) and Fe­(III), the EPR spectrum of which is governed by the ZFS and a predominantly isotropic g-tensor. High-field EPR and OS-ENDOR in these systems are known to provide valuable insights into metalloenzyme structures, including these ions, when ZFS axes are known.^32,55,56^ Furthermore, we demonstrated a good agreement of the experimentally determined ZFS principal axes with the simple electric-field gradient tensor and the Newman–Urban superposition approximations. Although accurate magnitudes of ZFS distributions remain difficult to reproduce computationally, the agreement in tensor orientations, particularly for **Gd-DO3A**, highlights the link between chelate geometry and ZFS anisotropy. As such, the experimentally derived ZFS PAS benchmarks will aid the development of quantum-chemical methods for the accurate prediction of ZFS tensors in lanthanide complexes.

## Experimental and Computational Details

### Synthesis and Sample Preparation

**1**, **2**, **3,** and **4** were synthesized and characterized according to earlier reports.^31,57,58^ The structure and purity of all complexes were confirmed using high-resolution mass spectrometry and ^1^H NMR spectroscopy prior to Gd­(III) complexation. Pulsed EPR and ENDOR spectra were recorded on 200 μM (**1**), 250 μM (**2**), and 300 μM (**3** and **4**) samples, containing 75:25 D_2_O:glycerol-d_8_ (v/v) (for **1** and **2**) and 50:50 D_2_O:glycerol-d_8_ (v/v) (for **3** and **4**).

The proteins **GB1-Q32C** and **Ub-M1C** were prepared as described previously^24^ and labeled with Gd­(III)-BrPSPyDO3A chelate (**Gd-DO3A** in Figure 1).^30^ They were dissolved in 25 mM D_2_O-based phosphate buffer (pD 7.0), 150 mM NaCl, with 20 vol % glycerol-d_8_ added as a cryoprotectant. The final concentrations of the proteins were 40 μM. The temperature-dependent spectra of **Gd-DO3A** were recorded for a **Gd-DO3A**-labeled **Ub T66C**, another variant of ubiquitin, the spectra of which were reported previously.^31^ The temperature-dependent spectra of **Gd-PyMTA** were recorded for complex **3**.

All samples were placed into 0.60 mm inner diameter fused silica tubes sealed with Critoseal.

### Spectroscopic Measurements

Pulsed EPR and ENDOR measurements of substances **1**–**4** and **Gd-DO3A**-labeled **Ub-M1C** were performed on a home-built W-band spectrometer equipped with a cylindrical TE_011_ cavity and a Helmholtz radiofrequency (RF) coil.^59^ The spectrometer has a 0–5 T cryogen-free magnet with an integrated variable temperature unit and a 300 mT sweep coil (J3678, Cryogenic Ltd.). It is equipped with a 2 W pulsed microwave power amplifier (QPP95023330-ZW1, Quinstar) and a pulsed 2 kW RF amplifier (BT02000-GammaS, TOMCO). The RF pulses were produced using an arbitrary-wave generator (DAx22000, Wavepond).

ENDOR spectra of **Gd-DO3A**-labeled **GB1-Q32C** at 2.5 K were recorded using a Bruker Elexsys E680 spectrometer (at the Max Planck Institute of Chemical Energy Conversion, MPI), equipped with a home-built W-band bridge employing a 2 W pulsed microwave power amplifier (QPP-94013338MPI, Quinstar), a closed-cycle 6 T split pair superconducting magnet with an integrated 2–300 K cryostat (J4233, Cryogenic Ltd.), and a 100 W RF amplifier (ZHL-100W-GAN+, Mini-Circuits).

Echo-detected electron paramagnetic resonance (ED-EPR) spectra were recorded using the Hahn echo (π/2 – τ – π – τ – *echo*) sequence. Mims ENDOR spectra were recorded using the sequence π/2 – τ – π/2 – T­(π_RF_) – π/2 – τ – *echo* – [τ_2_ – π – τ_2_ – echo – τ_2_ – (−π) – τ_2_ – echo]_n_ with a four-step phase cycle and a Carr–Purcell (CP) detection train at the end to enhance the signal-to-noise ratio.^60^ We used three to five CP echoes with τ_2_ = 600 ns for detection. Each echo was integrated over a 20 ns window, optimized for the best signal-to-noise ratio. Random sampling of RF was employed,^61^ with 5–10 shots acquired per frequency point in each scan. Microwave power was adjusted to give π pulses of 28–32 ns, using the Rabi nutation sequence, t_nut_ – t_wait_ – π/2 – τ – π – τ – *echo* (t_nut_ was varied; t_wait_ was chosen such as to let for the decay of the transverse magnetization). RF power was adjusted to yield the desired π_RF_ pulse length, using a Rabi nutation sequence π/2 – τ – π/2 – T­(t_RF_) – π/2 – τ – *echo*, with a constant mixing time *T* of 100 μs and varying RF pulse length, t_RF_. The RF pulse length was set to 35–60 μs. The mixing time *T* in the Mims ENDOR experiment was set to be 2–5 μs longer than the RF pulse length.

The ^19^F ENDOR spectra were baseline-corrected using linear or cubic polynomials, as shown in Figures S22, S23. A strong background slope at the high-frequency end of the spectra of **1** is due to the interference from high-spin electron manifolds to ^1^H ENDOR lines, as for this complex the Gd–F distance is the shortest, and ^19^F ENDOR spectra span the widest frequency range. The strong background features are observed outside the ^19^F spectrum and are not expected to alter the interpreted spectral shapes.

Electron spin–lattice relaxation times, ,*T*_1e_, were measured using the inversion recovery pulse sequence, π – t_wait_ – π/2 – τ – π – τ – *echo*, monitoring the echo intensity as a function of t_wait_. Electron spin phase memory times, *T*_M_, were measured using the Hahn echo sequence, π/2 – τ – π – τ – *echo*, following the echo intensity as a function of 2τ. The traces were fitted with stretched exponential functions. The relaxation parameters for all studied samples are summed up in Table S3; they were measured at 10 K at the field position corresponding to the maximum of the CT spectrum. It was previously shown that the spin–lattice relaxation is essentially independent of the field position within the spectrum, whereas the phase memory times are shorter off the CT due to transient fluctuations of the ZFS.^15^

### AIMD

For AIMD simulations, free **Gd-DO3A** containing a −SH group at the attachment site was used as the **Gd-DO3A** model, and complex **3** was used as the **Gd-PyMTA** model. The initial conformational search for the chelate structures was performed using CREST software (version 2.12) with GFN2-xTB and water as the solvent.^62^ Five lowest-energy conformations of the chelate shell, with slightly different geometries of the cyclen ring, were obtained for **Gd-DO3A**, and a single predominant conformation was found for the chelate structure of **Gd-PyMTA**. In each of these structures, a Y­(III) ion was substituted for Gd­(III), and explicit water molecules were added to complete the coordination shell: one for **Y-DO3A** and two for **Y-PyMTA**. The resulting structures were preoptimized using DFT (program package Orca 6.0.1,^63^ functional PBE,^64^ basis def2-SVP^65^ with a CPCM solvation model (H_2_O)^66^). For the structural alignment used for determining experimentally the ZFS PAS in the molecule only the DFT-optimized geometry with lowest-energy conformer of the chelate shell was used. In contrast, for the AIMD to calculations all 5 conformers of Y-DO3A obtained by CREST were used. Y­(III) possesses a very similar ionic radius, coordination geometry, and ligand charge distribution to Gd­(III), which dominate the EFG. Indeed, the DFT-optimized structures for Y and Gd chelates of both DO3A and PyMTA practically superimpose (Figure S13). Next, for each conformer, *ab initio* molecular dynamics (MD) simulations were carried out using the same DFT method. In each step of the calculation, the atomic forces acting on each degree of freedom were calculated from the DFT-computed energy gradient, and classical equations of motion were solved for the atomic positions and forces during the current time step. A coupled thermal bath ensures energy conservation. A propagation step of 1 fs was chosen for all simulations. The temperature was ramped from 10 to 160 K over 200 fs using a NHC thermostat with a time constant of 10 fs, and the MD trajectory was computed at 160 K using a NHC thermostat with a time constant of 30 fs.

In the resulting MD trajectories, the first 200 fs after the temperature ramp were discarded to allow for better equilibration, and only the MD snapshots corresponding to local energy minima were selected. For those, the EFG tensors were calculated using eq 11 and diagonalized, yielding the principal values and the corresponding PAS. The axes of the EFG tensor were ordered similarly to the ZFS tensor median values: |*V*_xx_| ≤ |*V*_yy_| ≤ |*V*_zz_|, to facilitate their comparison with ZFS tensors obtained from the EPR and ENDOR spectral simulations (cf. eq (S3), Supporting Information).

## Supplementary Material



## Notes

Reference frames and definition of angles; molecular geometry optimization and conformational analysis; temperature dependent ED-EPR spectra of Gd­(III) chelates and simulations; additional experimental and simulated ^19^F ENDOR spectra of **1** and **2**; molecular modeling and structural alignment of molecules **1**–**4**; ^1^H ENDOR data and simulations; comparison of optimized geometries for Gd­(III) and Y­(III) complexes; ZFS orientations calculated by CASSCF quantum chemistry calculations; EPR spectra of **Gd-DO3A** labeled proteins; additional simulations for the **Gd-DO3A** labeled proteins; background subtraction of ^19^F ENDOR spectra; electron spin relaxation of the studied samples (PDF)

## ∇

Department of Education, Indian Institute of Technology, Kharagpur 721302, WB, India

## ○

State Key Laboratory of Magnetic Resonance Spectroscopy and Imaging, National Center for Magnetic Resonance in Wuhan, Innovation Academy for Precision Measurement Science and Technology, Chinese Academy of Sciences, Wuhan, 430071 P. R. China
