# Supplementary material for: Unlocking Gd(III) Anisotropy: Determining the Zero-Field Splitting Axes to Enhance Spin-Label Structural Analysis
Source: J Am Chem Soc. 2026 Jun 2;148(23):23597–610. doi: 10.1021/jacs.5c22555 (PMC13281530; doi:10.1021/jacs.5c22555)
Supplement: Supplementary file 1 [file ja5c22555_si_001.pdf]

Supporting information

Unlocking Gd(III) Anisotropy: Determining Zero-Field Splitting Axes to Enhance Spin-Label Structural Analysis

Alexey Bogdanov[1], Veronica Frydman[2], Xun-Cheng Su[3], Manas Seal[1#], Wenkai Zhu[4§], Angela M. Gronenborn[4], Alexander Schnegg[5], and Daniella Goldfarb[1*]*

[1] Department of Chemical and Biological Physics, The Weizmann Institute of Science, P. O. Box 26, Rehovot, 7610001, Israel

[2] Department of Chemical Research Support, The Weizmann Institute of Science, P. O. Box 26, Rehovot, 7610001, Israel

[3] State Key Laboratory of Elemento-Organic Chemistry, Nankai University, Tianjin 300071 P. R. China

[4] Department of Structural Biology, University of Pittsburgh, 4200 Fifth Ave, Pittsburgh, PA 15260, United States

[5] Max Planck Institute for Chemical Energy Conversion, 34-36 Stiftstraße, Mülheim an der Ruhr, 45470, Germany

[#] Current address: Department of Education, Indian Institute of Technology, Kharagpur 721302, WB, India

[§] Current address: State Key Laboratory of Magnetic Resonance Spectroscopy and Imaging, National Center for Magnetic Resonance in Wuhan, Innovation Academy for Precision Measurement Science and Technology, Chinese Academy of Sciences, Wuhan, 430071 P. R. China

[*]Corresponding authors

E-mails: daniella.goldfarb@weizmann.ac.il, alexey.bogdanov@weizmann.ac.il

Contents

S1. Reference frames and definition of angles S2
S2. Molecular geometry optimization and conformational analysis S3
S3. Temperature dependent ED-EPR spectra of Gd(III) chelates and simulations S6
S4. Additional experimental and simulated ^{19}F ENDOR spectra of 1 and 2. .... S8
S5. Molecular modeling and structural alignment of molecules 1–4 .... S11
S6. ^{1}H ENDOR data and simulations S13
S7. Comparison of optimized geometries for Gd(III) and Y(III) complexes S17
S8. ZFS orientations calculated by CASSCF quantum chemistry calculations........ S18
S9. EPR spectra of Gd-DO3A labeled proteins S19
S10. Additional simulations for the Gd-DO3A labeled proteins. S20
S11. Background subtraction of ^{19}F ENDOR spectra........ S28
S12. Electron spin relaxation of the studied samples S30
Supplementary references S31

S1. Reference frames and definition of angles

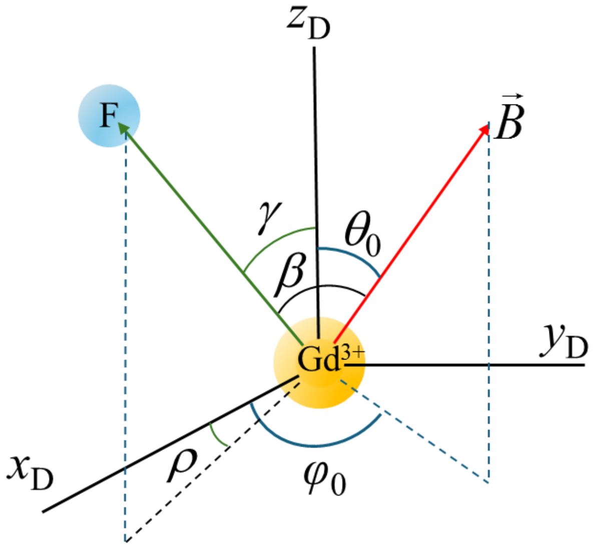

Fig. S1. Definition of angles used throughout this work. x_D, y_D, z_D – principal-axes of the ZFS tensor; θ_0 and φ_0 are polar and azimuthal angles, respectively, defining the orientation of the external magnetic field; γ and ρ are polar and azimuthal angles, respectively, defining the orientation of the Gd–F dipolar vector; β is the angle between the magnetic field and the Gd–F vector.

S2. Molecular geometry optimization and conformational analysis

Geometrical structures of **1**–**4** were optimized using density functional theory (DFT, Orca software version 6.0.1,[S1] functional PBE0[S2] with D3(BJ) dispersion correction,[S3] basis def2-TZVPP on Gd and def2-SVP on all other atoms[S4] and the CPCM solvation model (H_2O)[S5]). One (for **Gd-DO3A** chelate) and two (for **Gd-PyMTA** chelate) water ligands were added to complete the coordination shell of Gd(III). The Hessian matrices were computed at equilibrium to confirm that the DFT-derived geometries constitute energy minima. Gibbs free energies were computed at 160K (estimated glass transition point for water-glycerol mixtures), using an ideal gas approximation.[S6, 7]

1 was found to be rigid, having a single conformation. **2** comprises a thioether bond, rotation around which leads to two possible rotamers (**Fig. S2**). However, as the energy of one rotamer is ~13 kJ/mol higher than the other; it is not expected to contribute significantly to the spectral properties of **2** in the frozen solution, and was not considered in simulations.

For **3**, the piperidine group exists predominantly in a chair configuration, with the fluorine substituent either axial or equatorial. The energies of these isomers are identical within the computational error (**Fig. S3**), and the Gd–F distances and vector orientations differ by less than 0.1 Å and 5–7º, respectively. Essentially, these differences are within experimental uncertainty, and, for practical reasons, only the axial isomer was used.

For **4,** two possible conformations exist, where the 4-fluorinated phenyl ring is either perpendicular to the pyridyl group of PyMTA, or parallel to it (**Fig. S4**), with the first conformer being ~1 kJ/mol lower than the second. The Gd-F distance in the first conformer (13.6 Å) is much closer to the one obtained experimentally (13.2 Å), compared to the calculated second one (11.6 Å). Therefore, we posit that the first variant is predominantly populated and contributes the most to the ENDOR spectra.

In summary, the above reasoning shows that for each model complex only one conformer should be considered.

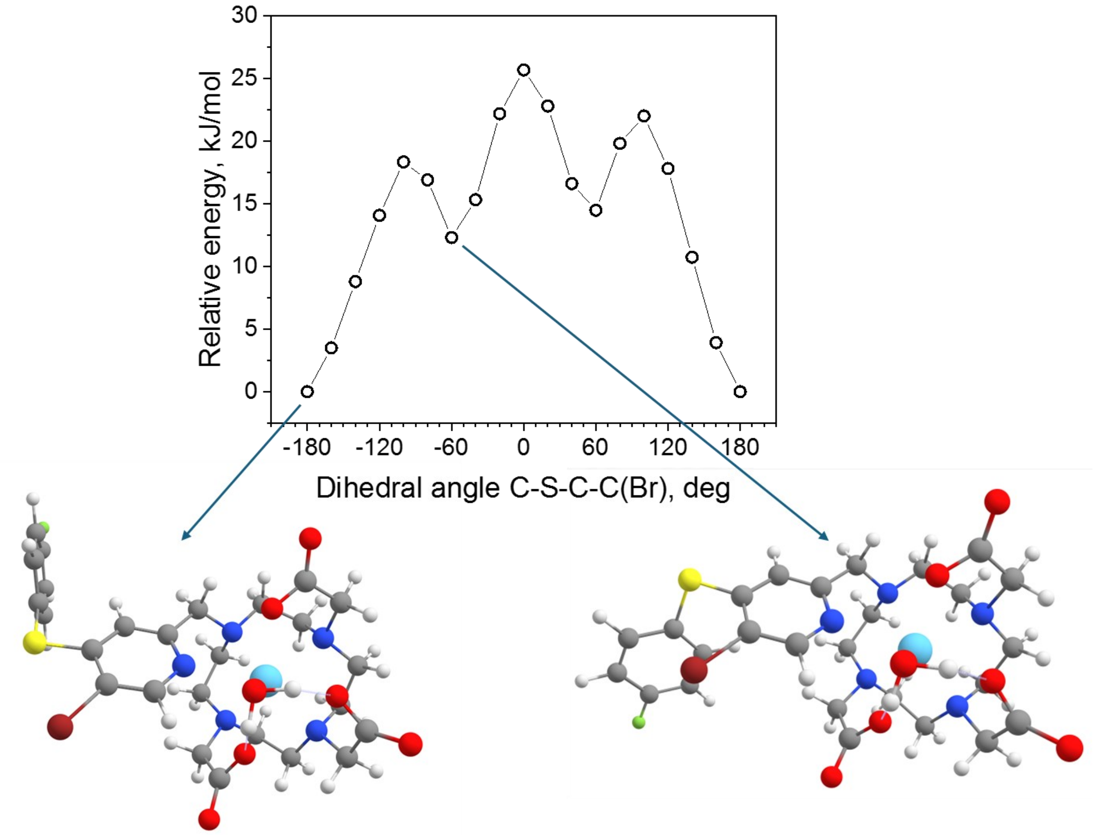

Fig. S2. Relaxed energy scan profile from DFT calculations for **2** for rotation around the C–S bond. The structures of the two conformations depicted below the graph correspond to the indicated energy minima.

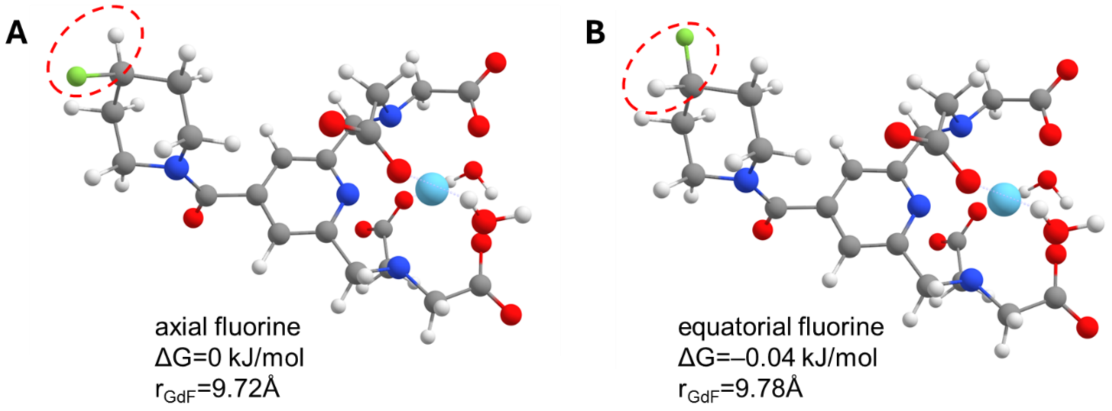

Fig. S3. Structures for the two isomers of **3**, with the fluorine atom occupying axial (**A**) and equatorial (**B**) positions in the piperidine ring. The relative Gibbs free energies and Gd–F distances are listed below the structures.

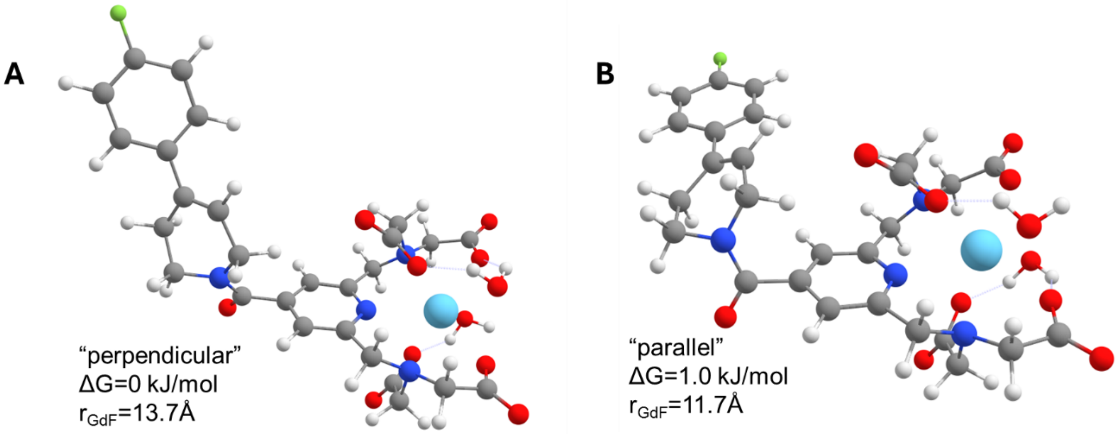

Fig. S4. Structures of the two conformers of **4**, with the 4-fluorophenyl ring positioned approximately perpendicular (**A**) and parallel (**B**) to the pyridine group of PyMTA. The relative Gibbs free energies and Gd–F distances are listed next to the structures.

S3. Temperature dependent ED-EPR spectra of Gd(III) chelates and simulations

ED-EPR simulations

ED-EPR spectral simulations were carried out using a previously published approach and home-written software based on the spin Hamiltonian given by eq. (4) (*main text*).[S8, 9]

The spectra recorded at different temperatures were jointly simulated, and the Boltzmann populations of the electron spin levels at each temperature were considered. Flip angle correction to account for the different nutation frequencies of the electron spin transitions was implemented, as described previously,[S8] . The ZFS parameters *D* and *E* were assumed to exhibit uncorrelated Gaussian distributions, with probability densities given by

$$P(D)=\sqrt{\frac{2}{\pi\cdot\Delta D^2}}\cdot\exp\left[-\frac{2(D-D_0)^2}{\Delta D^2}\right],P(E)=\sqrt{\frac{2}{\pi\cdot\Delta E^2}}\cdot\exp\left[-\frac{2(E-E_0)^2}{\Delta E^2}\right] \quad \text{(S1)}$$

where D_0 and E_0 are the median values and ΔD and ΔE are distribution widths. Conventionally, the ZFS principal components of are ordered as follows:

$$|D_{xx}|\le|D_{yy}|\le|D_{zz}| \quad \text{(S2)}$$

In the presence of a broad distribution of ZFS parameters, reordering the assignment of ZFS principal values according to eq. (S2) for each component can lead to different orientations of ZFS axes in the molecular frame. To prevent this possibility, here we assume that *D* and *E* are formal parameters possessing independent Gaussian distributions. As a result, D_{xx}, D_{yy} and D_{zz}, being linear combinations of *D* and *E* (*cf.* eq. (2) in the *main text*), also possess Gaussian distributions. However, for consistency, the median values of *D* and *E* distributions were chosen such as to satisfy the condition: $0\le E_0/D_0\le 1/3$, and thus, the median principal values of ZFS would satisfy the condition:

$$|D_{xx,0}|\le|D_{yy,0}|\le|D_{zz,0}| \quad \text{(S3)}$$

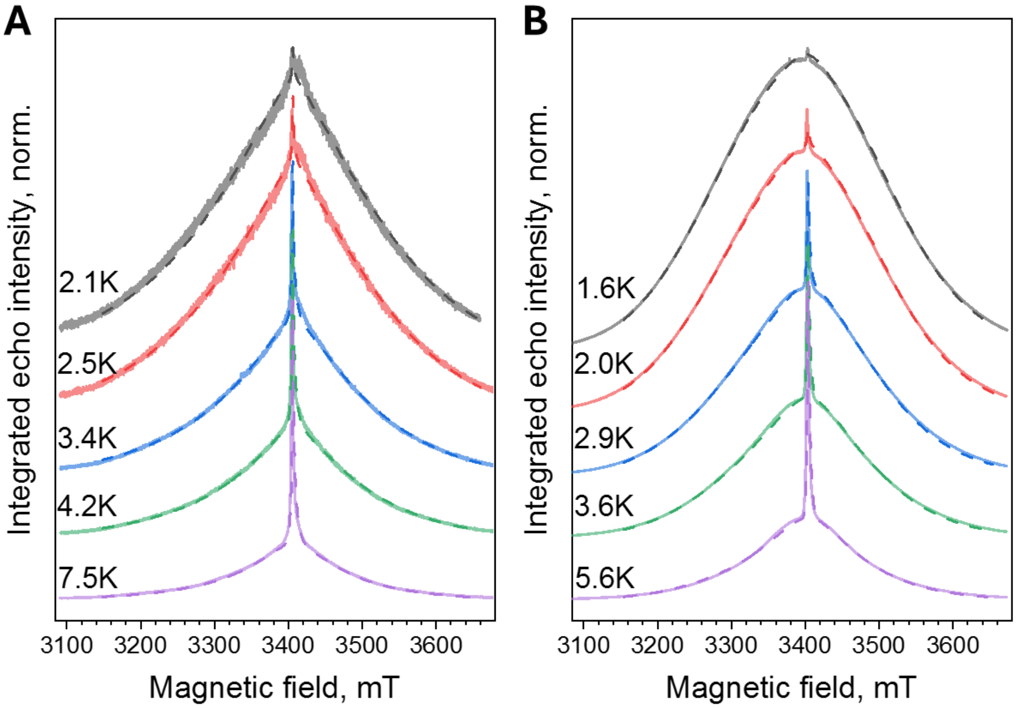

Fig. S5. Temperature dependence of W-band ED-EPR spectra of **Gd-DO3A** (in 40 µM labeled 4tFm-Phe45 **Ub-T66C**[S8]) (**A**), and 300 µM solution of **Gd-PyMTA** (in **3)** (**B**). Solid lines represent the experimental spectra and dashed lines the simulation. All spectra for a particular sample were simulated jointly with the same *D* and *E* distributions.

S4. Additional experimental and simulated ^{19}F ENDOR spectra of 1 and 2.

OS-ENDOR simulations

^{19}F OS-ENDOR spectra were simulated using previously described approaches and home-written software.[S8, 9] The ENDOR resonance frequencies were calculated using eqs. (7)–(9) (*main text*). The signal intensity at each frequency was calculated as a product of the three contributions:

(a) The relative EPR intensity of the $|m_S\rangle \leftrightarrow |m_S+1\rangle$ transition at each field. This was pre-calculated during the ED-EPR spectra simulations and further optimized to best fit the ^{19}F ENDOR spectra, as previously described;[S8]

(b) The probabilities of the ZFS orientations θ_0 and φ_0 at each field for each electron spin transition. These were pre-calculated during ED-EPR spectra simulations.

(c) The Mims ENDOR blind-spot pattern, according to the well-known expression:[S10]

$$F_{\mathrm{ENDOR}} \propto \sin^2\left[\pi \cdot a(\beta)\tau\right] \quad \text{(S4)}$$

The experimental spectra recorded at different field positions were jointly simulated using an adaptive non-linear least-squares algorithm.[S11] The uncertainties of the obtained angles γ and ρ were estimated by incrementally shifting each angle from its optimal value and monitoring the resulting increase in the objective function (the sum of squared deviations between experimental and simulated spectra). The uncertainty was defined as the shift at which the objective-function increase exceeded the experimental noise level, determined from the tails of the ENDOR spectra.

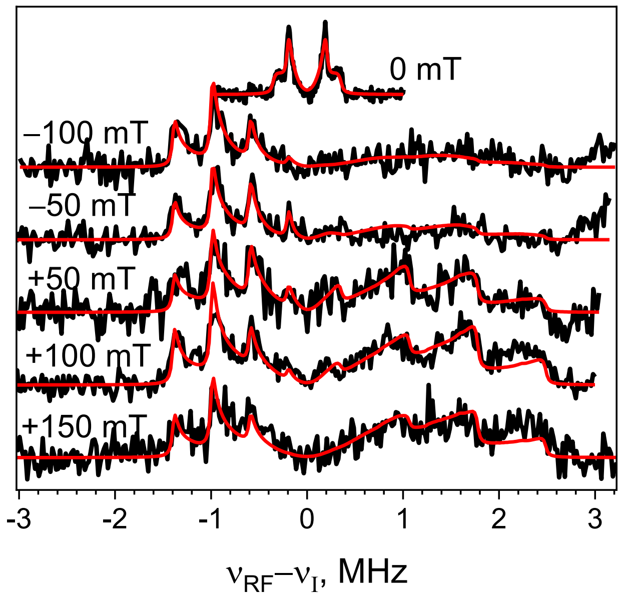

Fig. S6. W-band Mims ^{19}F OS-ENDOR spectra of **1** (black lines), recorded using an interpulse delay in the Mims sequence of τ=600 ns, and their simulations (red lines). The spectra were simulated jointly and with the same parameters as those recorded with τ=1000 ns shown in **Fig. 3A** of the *main text*.

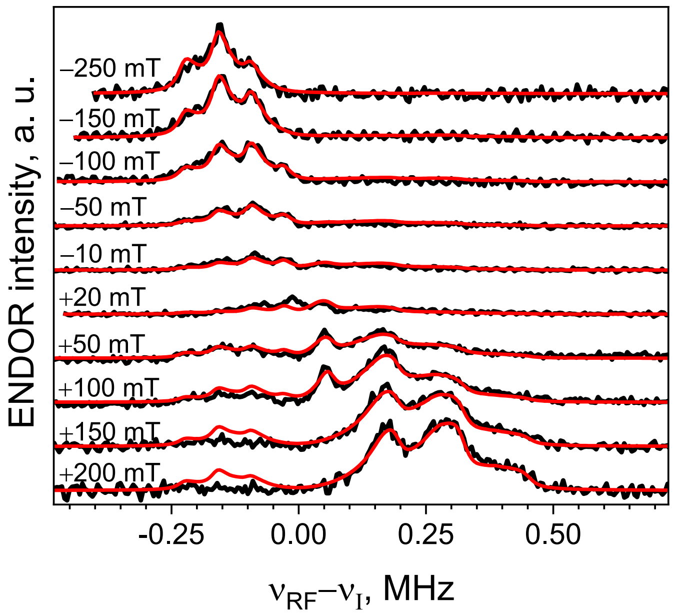

Fig. S7. Experimental (black lines) and simulated (red lines) W-band Mims ^{19}F OS-ENDOR spectra of **2**, recorded off the Gd(III) CT, using an interpulse delay in the Mims sequence of τ=2000 ns. The full field dependence of the ENDOR spectra is provided in this figure, with six selected spectra shown **Fig. 3B** of the *main text*.

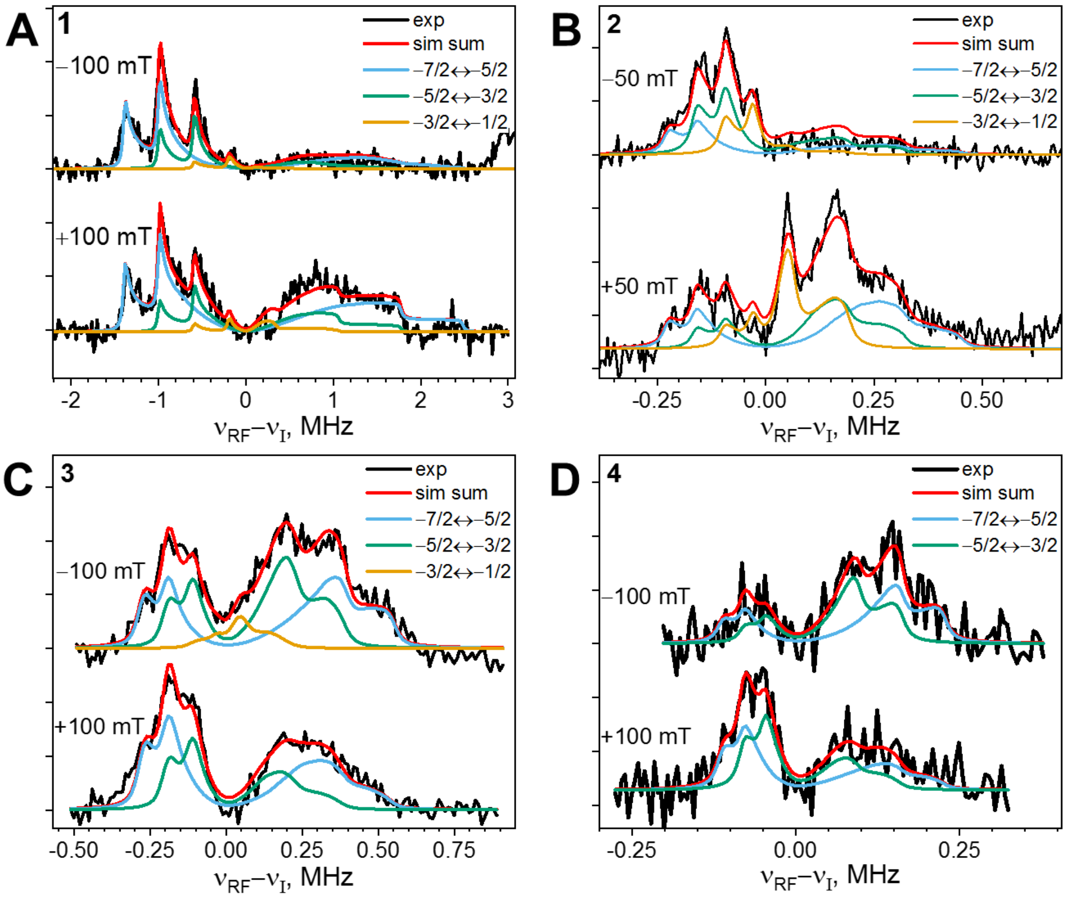

Fig. S8. Experimental (black lines) and simulated (red lines) ^{19}F OS-ENDOR spectra of **1** (**A**), **2** (**B**), **3** (**C**) and **4** (**D**), respectively, with the contributions of the individual EPR transitions plotted in colored lines. The spectra were recorded at 4 K with $\tau = 1$ μs (for **1**) and $\tau = 2$ μs (for **2–4**).

S5. Molecular modeling and structural alignment of molecules 1–4

Optimized structures for each pair of model complexes with the same chelate type were superimposed using the PyMOL software,[S12] and the origin of the coordinate frame was placed at the Gd(III) ion. This produces a combined molecular structure with both fluorine atoms positioned relative to the same Gd chelate.

We then determined the ZFS PAS position in the molecular structure, consistent with the experimental ^{19}F OS-ENDOR data. This entailed finding an optimal rotation matrix using the Kabsch algorithm,[S13] that positioned the fluorine atoms in the combined model as close as possible to the experimentally determined fluorine positions, F_j in ZFS PAS, given by:

$$F_j = \left(\pm r_j \cos\rho_j \sin\gamma_j, \quad \pm r_j \sin\rho_j \sin\gamma_j, \quad \pm r_j \cos\gamma_j \right), \tag{S5}$$

where r_j, γ_j and ρ_j (j=1,2) are the polar coordinates of the fluorine atoms in the model complex (**1** and **2** for **Gd-DO3A**, **3** and **4** for **Gd-PyMTA**). The "±" in eq. (S5) originates from the orthorhombic symmetry of the spin Hamiltonian (eq. (1), *main text*), resulting in symmetrical positions across the ZFS coordinate planes, which cannot be distinguished by the OS-ENDOR data. Only pairs of F_j vectors for which the angle F_1-Gd-F_2 was close to the one in the molecular structure were considered. For **Gd-DO3A** this angle is 42°, as determined by ENDOR (vs. 56° in the combined DFT-optimized structure, see **Fig. S9**), and for **Gd-PyMTA** this angle is 20° from ENDOR (vs. 44° for the DFT-optimized structure). We thus ended up with two final non-equivalent ZFS PAS orientations that cannot be distinguished based on the experimental ^{19}F OS-ENDOR spectra of the two model complexes alone.

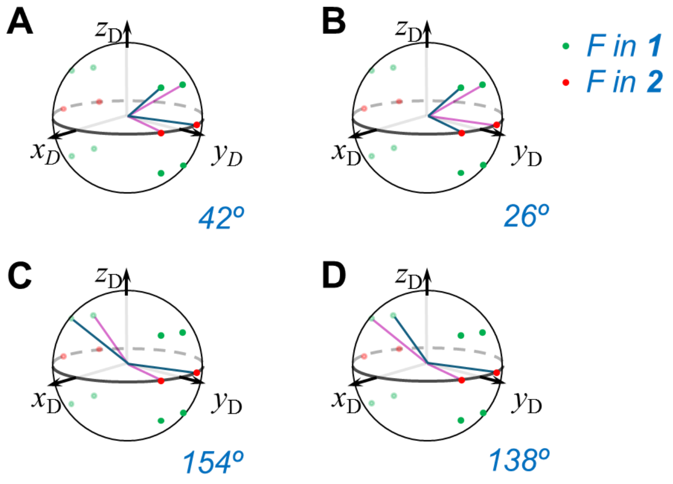

Fig. S9. Illustration of orientations of ZFS tensor axes on a sphere for **1** and **2** that produce the same ENDOR spectra. The coordinate system corresponds to the ZFS PAS, with the Gd atom at the origin. Green points on a unit sphere represent possible locations of the Gd-F_1 vector on the sphere (F in compound **1**, eight possible orientations), and red points represent the Gd-F_2 vector (F in complex **2,** because γ_2=90º, there are only four possible orientations). **A-D** show different combinations of inequivalent pairs of vectors, presented by blue and magenta lines on each panel, that generate different values of the F_1–Gd–F_2 angle, as listed below each sphere.

S6. [1]H ENDOR data and simulations

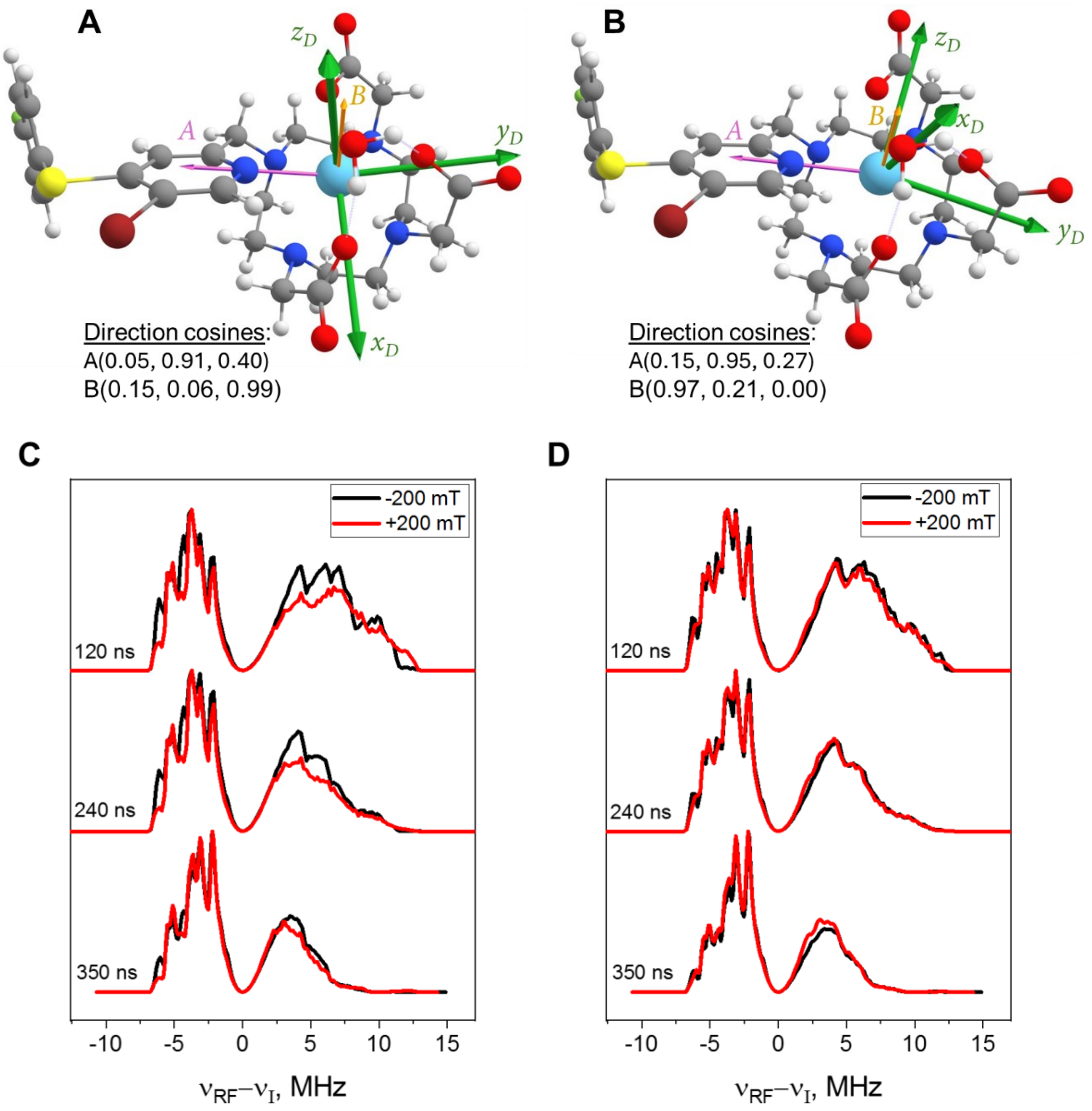

Fig. S10. (**A, B**) Optimized structures of **2** with the two alternative orientations of the ZFS PAS, both matching the experimental ^{19}F OS-ENDOR data. Magenta and yellow arrows depict the molecular vectors A and B, respectively, and the absolute values of direction cosines defining these vectors in ZFS PAS are listed. (**C, D**) ^{1}H OS-ENDOR spectra predicted for the structures in (**A**) and (**B**), using the hydrogen positions listed in **Table S1**.

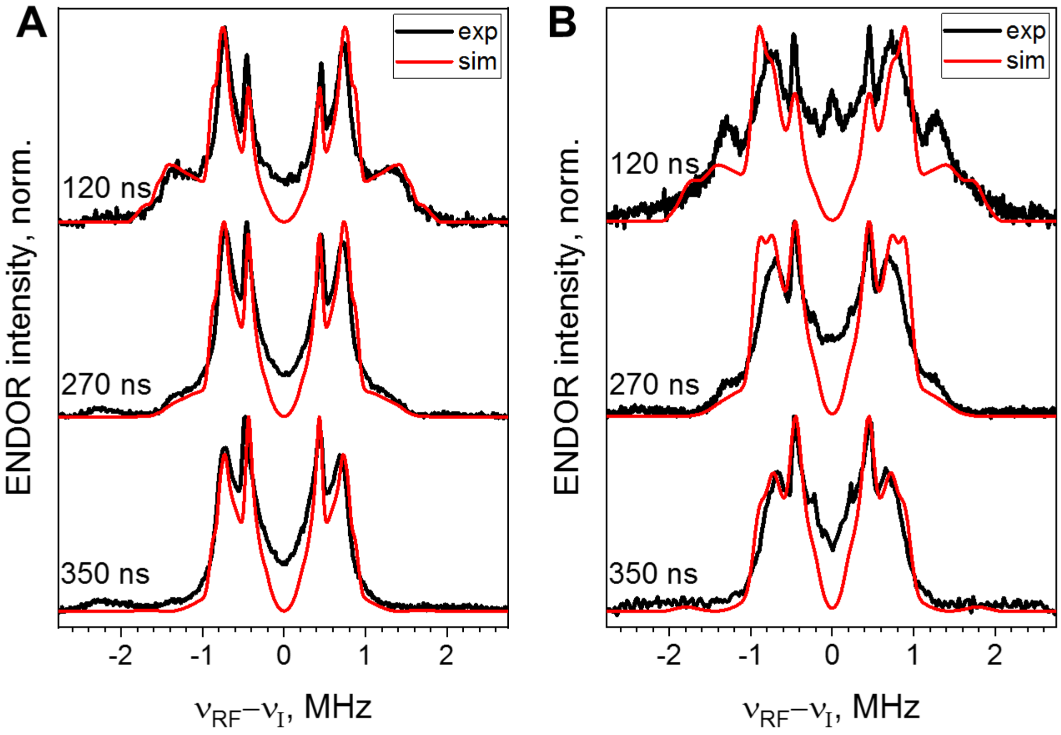

Fig. S11. Experimental (black lines) and simulated (red lines; no fitting parameters used) ^{1}H Mims ENDOR spectra of the free **Gd-DO3A** spin label (**A**) and complex **3** (**B**), recorded at the CT for different values of the interpulse delay τ. The hydrogen positions from the DFT optimized molecular structures (listed in **Table S1**) were used for the simulation.

Table S1. Geometrical parameters for protons surrounding the Gd(III) ion in spin label **Gd-DO3A** and complex **3** (comprising **Gd-PyMTA** chelate), obtained from DFT optimized structures: Gd–H distances (r_{GdH}), hyperfine dipolar coupling between Gd(III) and H ($a_{\perp}$) and polar and azimuthal angles (γ and ρ in degrees) defining the positions of the corresponding protons in the ZFS PAS, for the ZFS alignments presented in **Figs. S10A,B** and **S12A,B**.

**Gd-DO3A**						**Gd-PyMTA**					
r_{GdH}, Å	$a_{\perp}$, kHz	structure 1[a]		structure 2[b]		r_{GdH}, Å	$a_{\perp}$, kHz	structure 1[c]		structure 2[d]	
		γ	ρ	γ	ρ			γ	ρ	γ	ρ
4.51	0.863	47.2	69.9	58.0	25.5	5.57	0.457	77.1	63.9	36.4	75.9
3.75	1.497	68.9	76.5	58.4	51.8	5.55	0.461	33.0	79.9	65.2	55.9
3.79	1.453	30.4	63.1	88.5	12.3	3.46	1.903	36.1	33.9	78.8	59.5
4.48	0.877	52.6	69.6	87.9	34.9	4.42	0.915	15.0	52.0	86.8	42.4
4.46	0.887	37.2	22.9	46.0	13.3	3.53	1.798	78.3	44.8	24.6	16.2
3.67	1.594	15.4	36.1	68.4	12.5	4.43	0.909	76.8	67.6	44.1	36.3
3.69	1.568	57.9	14.9	39.3	63.8	3.40	2.002	44.5	25.7	51.6	29.4
4.45	0.896	37.9	29.4	59.2	49.3	4.31	0.989	30.3	56.4	69.6	12.7
4.44	0.904	41.4	82.5	88.7	58.3	4.35	0.961	17.3	17.0	71.9	15.0
3.76	1.489	19.3	84.6	89.3	35.4	3.71	1.552	35.5	54.2	80.3	8.0
3.66	1.610	67.2	69.7	58.9	75.7	3.50	1.843	42.4	37.5	54.3	19.5
4.44	0.903	49.1	53.7	58.2	49.1	4.35	0.956	40.1	72.6	68.1	1.7
3.70	1.562	75.5	17.0	40.6	40.2	4.30	0.994	72.8	80.9	74.3	42.6
4.45	0.894	55.4	28.5	60.3	25.5	3.78	1.466	54.0	63.7	87.5	26.4
4.43	0.911	51.3	10.5	46.2	10.0						
3.66	1.607	28.8	4.4	69.0	10.9						
3.78	1.457	82.0	46.7	28.8	68.4						
4.43	0.908	65.6	29.6	20.7	14.2						
3.54	1.782	79.4	42.0	59.0	80.4						
4.36	0.953	66.3	62.1	77.9	84.9						
3.57	1.729	89.5	46.0	28.9	84.2						
4.34	0.965	78.3	25.7	20.4	40.7						
4.43	0.906	80.2	57.3	76.5	60.4						
3.52	1.817	83.5	42.0	59.3	75.7						
3.48	1.874	50.5	47.7	54.2	46.1						
5.54	0.464	84.6	75.9	87.1	78.1						

[a] For the structure shown in **Fig. S10A**
[b] For the structure shown in **Fig. S10B**
[c] For the structure shown in **Fig. S12A**
[d] For the structure shown in **Fig. S12B**

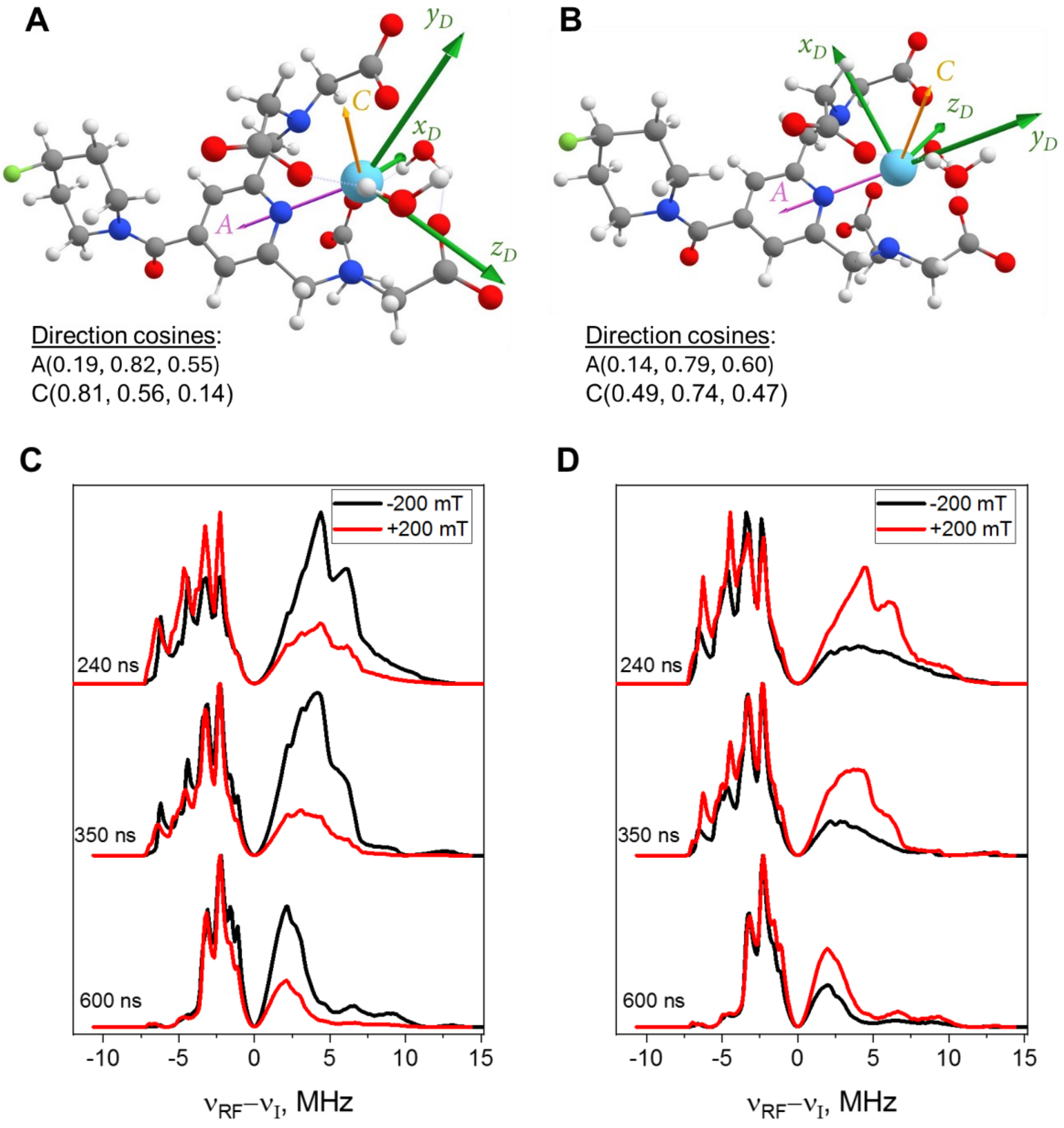

Fig. S12. (**A, B**) Optimized structures of **3** with the two alternative orientations of the ZFS PAS, both matching the experimental ^{19}F OS-ENDOR data. Magenta and yellow arrows depict the molecular vectors A and C, respectively, and the absolute values of direction cosines defining these vectors in ZFS PAS are listed. (**C, D**) ^{1}H OS-ENDOR spectra predicted for the structures in (**A**) and (**B**), using the hydrogen positions listed in **Table S1**.

S7. Comparison of optimized geometries for Gd(III) and Y(III) complexes

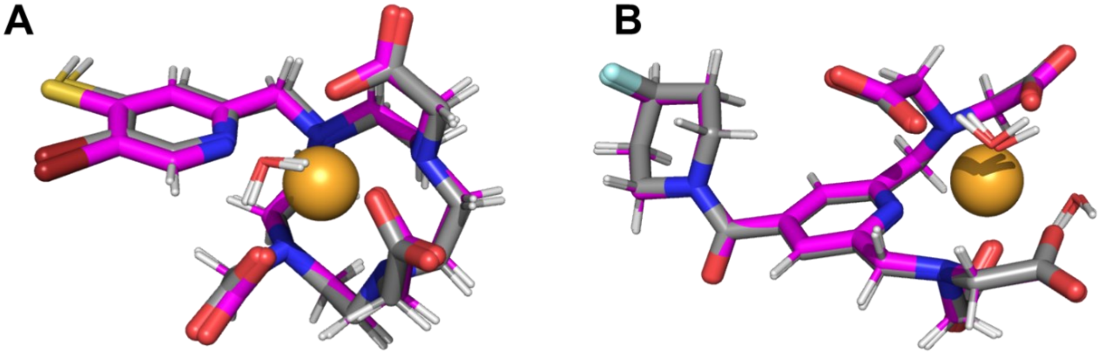

Fig. S13. Superposition of the DFT-optimized structures of **Gd-DO3A (**free label**)** and **Y-DO3A** (**A**) and **Gd-PyMTA** and **Y-PyMTA** (complex **3)** (**B**). The backbone of the Gd(III) structures is shown in gray, and that of the Y(III) structures in magenta. The superimposed Gd/Y ions are shown as orange spheres.

S8. ZFS orientations calculated by CASSCF quantum chemistry calculations

ZFS tensors were computed *ab initio* for optimal geometries of **Gd-PyMTA** and **Gd-DO3A** using the complete active space self-consistent field (CASSCF) as implemented in Orca 6.0.1. The active space comprised the seven electrons in seven 4*f*-like orbitals. State-averaged CASSCF calculations were performed over a manifold of low-lying spin states (multiplicities 8, 6, 4 with 1, 42, 21 roots each). Dynamic correlation was included using N-electron valence state second-order perturbation theory (NEVPT2).[S14] Scalar relativistic effects were treated using the exact two-component (X2C) Hamiltonian.[S15] ZFS tensors were computed using an effective Hamiltonian technique.

The obtained ZFS magnitudes were found: for **Gd-DO3A** D=3660 MHz, E/D=0.29, for **Gd-PyMTA** D=3690 MHz, E/D=0.25; thus the computed D values are 2–3 times larger than the experimentally obtained ones. The obtained ZFS PASs are shown in **Fig. S14**, together with their defining direction cosines of the structural vectors A, B and C.

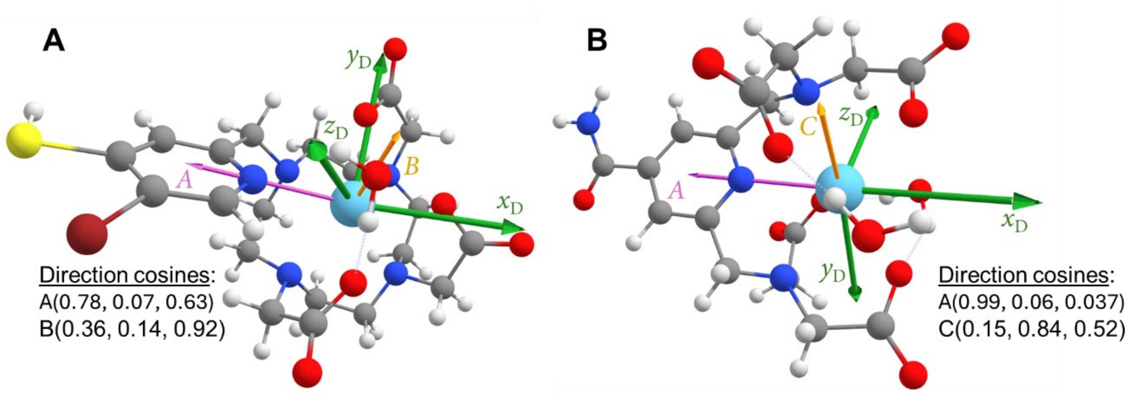

Fig. S14. Orientation of ZFS tensors obtained by multireference quantum chemistry computation for optimal geometries of **Gd-DO3A** (**A**) and **Gd-PyMTA** (**B**). ZFS PAS are shown as green arrows. Magenta and yellow arrows depict the molecular vectors A, B and C, and the absolute values of direction cosines defining these vectors in ZFS PAS are listed in each panel. For the calculations using EFG tensors and superposition model, the direction cosines are: for **Y-DO3A** A(0.87, 0.12, 0.47), B(0.15, 0.09, 0.98), for **Y-PyMTA** A(0.83, 0.46, 0.3), C(0.19, 0.65, 0.73).

S9. EPR spectra of Gd-DO3A labeled proteins

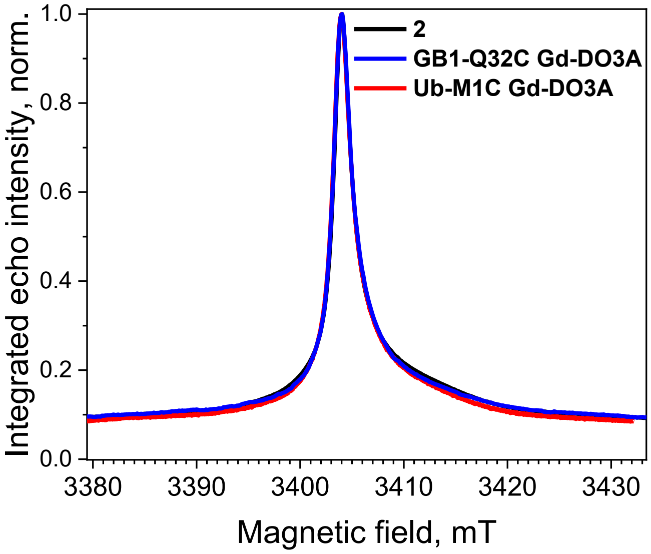

Fig. S15. CT region of echo-detected EPR spectra of complex **2** (black lines) and Gd-DO3A labeled proteins, **GB1-Q32C** (blue line) and **Ub-M1C** (red line).

S10. Additional simulations for the Gd-DO3A labeled proteins.

^{19}F OS-ENDOR spectra calculated using the *in silico* predicted rotamer ensemble of **Gd-DO3A** attached to **GB1-Q32C** were obtained as follows. Each of the 200 rotamers produced by the MtsslSuite software[S16] was superimposed on the optimized structure of **Gd-DO3A** in the ZFS PAS (**Fig. 5A**; *main text*) using the Horn quaternion method.[S17] As a result of this superposition, the ZFS axes directions relative to the rotamer structure were found, and the polar coordinates of the Gd–F vector, r_j, γ_j and ρ_j, were calculated. The distributions of values for these parameters are provided as histograms in **Fig. S16C** and were used to simulate the ^{19}F OS-ENDOR spectra shown in **Fig. S16D**. A Lorentzian linewidth of 5 kHz was used in the ENDOR simulations.

The same procedure was carried out using the MMM software,[S18] which assigns relative populations based on precomputed rotamer libraries and estimated interactions with the protein backbone (**Fig. S17**). Incorporating these population shifts the predicted Gd–F distance distributions toward shorter distances, in disagreement with the experimental ENDOR spectra (**Fig. S17A,B**). Assuming equal probabilities for all rotamers (**Fig. S17C,D**) improves the match with the experimental splittings; however, neither approach reproduces the experimental orientation-selection patterns.

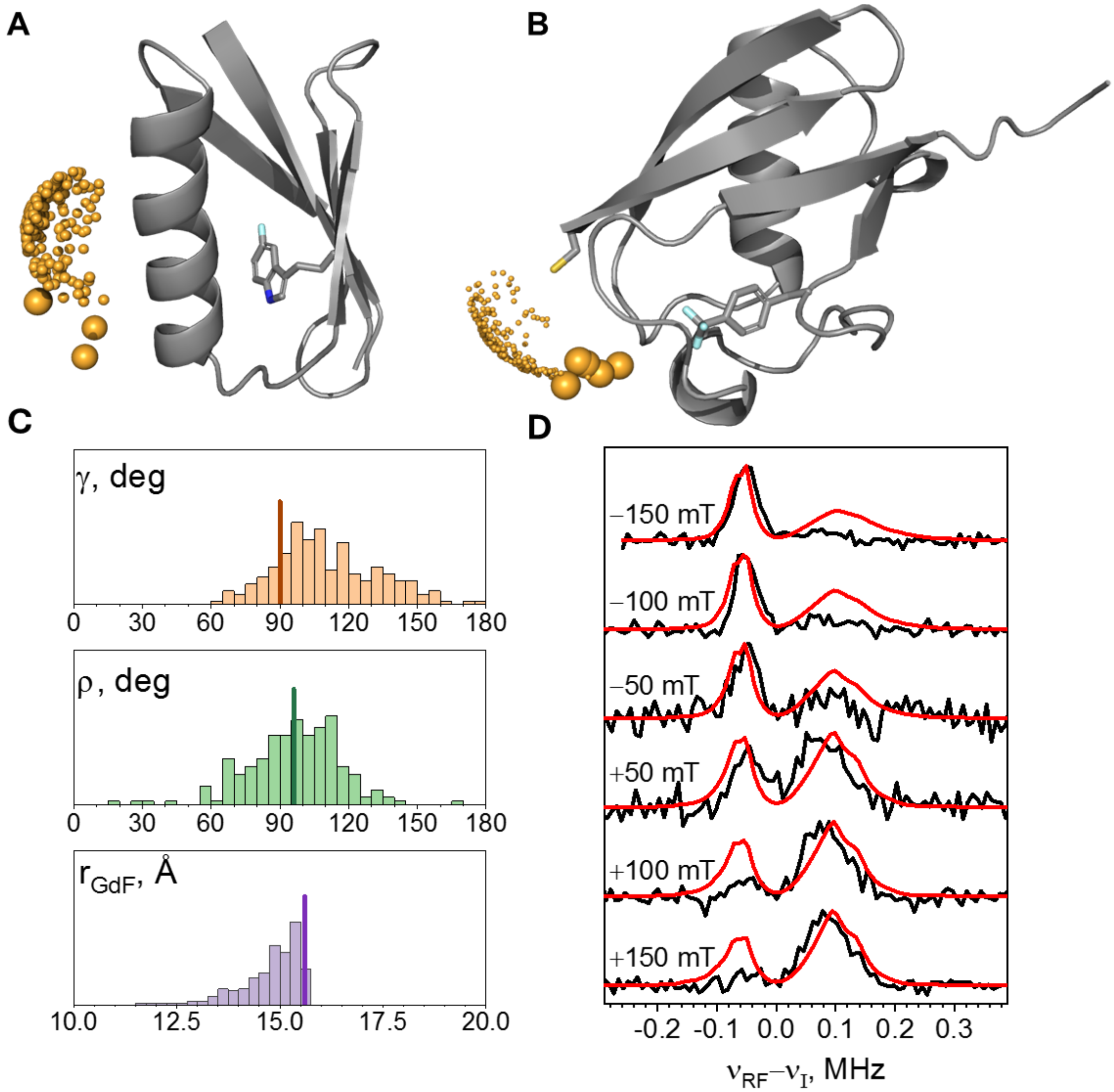

Fig. S16. Structures of proteins 5F-Trp 43 **GB1-Q32C** (**A**) and 4tFm-Phe45 **Ub-M1C** (**B**) in ribbon representation (based on the coordinates of PDB ID 1GB1[S19] and 1UBQ,[S20] respectively). The fluorinated amino acids are shown in stick representation, and the ^{19}F OS-ENDOR derived Gd(III) ions as large yellow spheres. The small spheres represent possible Gd(III) positions derived using MtsslSuite software. (**C**) Histograms of the distributions of the polar coordinates *r*, *γ* and *ρ* of the Gd–F vector in ZFS PAS of Gd(III), calculated for rotamers by MtsslSuite for 5F-Trp43 **GB1-Q32C**. Thick vertical lines reflect the values of *r*, *γ* and *ρ* determined from ^{19}F OS-ENDOR experiments. (**D**) Simulation of ^{19}F OS-ENDOR spectra based on the distributions shown in panel (**C**). Simulations of the ENDOR spectrum recorded at the central transition with the distance distribution shown can be found in Fig. 2 in reference S21.

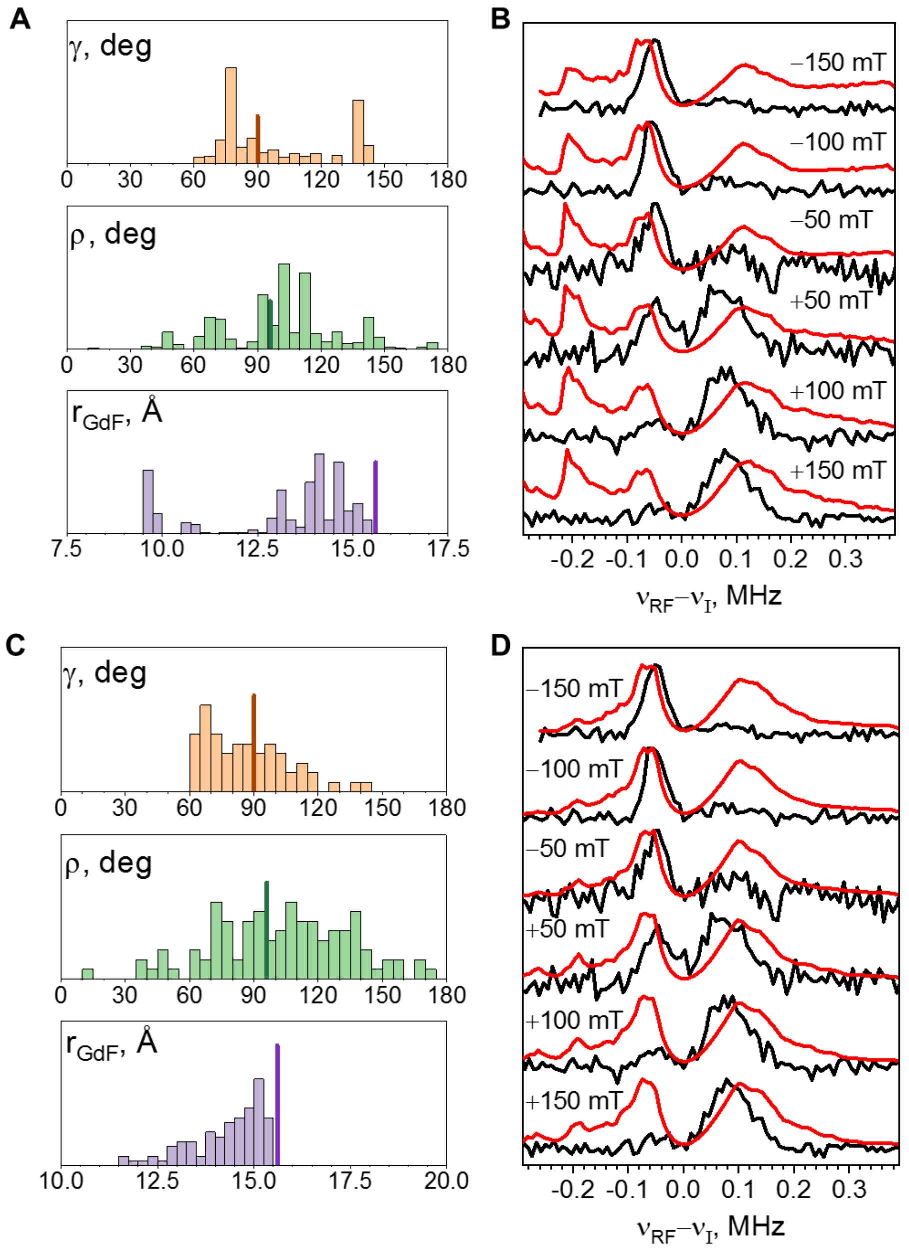

Fig. S17. (**A, C**) Histograms of the distributions of the polar coordinates r, γ and ρ of the Gd–F vector in ZFS PAS of Gd(III), calculated for rotamers obtained by MMM for 5F-Trp43 **GB1-Q32C**. Thick vertical lines reflect the values of r, γ and ρ determined from ^{19}F OS-ENDOR experiments. (**B, D**) Simulation of ^{19}F OS-ENDOR spectra based on the distributions shown in panels (**A, C**), respectively. In panels (**A, B**) rotamer populations provided by MMM were used, and in panels (**C, D**) all found rotamers were considered equiprobable. For the simulated spectra in panel (**B**), the parallel components of the short-distance contribution fall beyond the frequency scale of the graph and the experimentally recorded spectra.

The calculated conformer distributions can be used to quantify the restrictions on the conformation space of the label imposed by the experimental ENDOR constraints, both with and without including the orientation information. This is illustrated in **Table S2**, which reports the number of spin label conformers generated by MtsslSuite that (i) satisfy the experimental Gd–F distance and (ii) simultaneously satisfy both distance and orientation constraints. Distance agreement was defined using a ±2 Å cutoff, consistent with prior estimates of distance distribution width based on ENDOR spectral width.[S21] Agreement with the orientation constraints was assessed using the experimental uncertainties in the angles γ and ρ determined in the present work. The data in **Table S2** show that including ENDOR derived angular information imposes substantially more restrictions on the allowed conformational space than applying the distance criterion alone.

Table S2. Reduction of predicted conformation space of the spin label based on the experimental ENDOR constraints.

System	number of spin label conformers		
	total	constrained r[a]	constrained r, γ, ρ[b]
**GB1-Q32C Gd-DO3A**	201	184	45
**Ub-M1C Gd-DO3A**	201	39	0[c]

[a] conformers, for which the Gd–F distance matches the experimental one within ±2 Å
[b] conformers, for which in addition angles γ and ρ match the experimentally obtained within experimental uncertainties (listed in **Table 2**, *main text*).
[c] Zero conformers found for Ub-M1C Gd-DO3A is a consequence of structural changes arising from the attachment of the spin label as reported earlier.[S21]

Placement of the spin label based on ^{19}F OS-ENDOR data

Positioning of the **Gd-DO3A** label based on the ^{19}F OS-ENDOR experimental constraints was performed as outlined in **Fig. S18**. First, the experimental fluorine positions in the ZFS PAS were calculated using eq. (S5), yielding the fluorine geometry in the ZFS PAS (*step 1*). The label was then positioned relative to the protein structure such that the fluorine coordinates matched those obtained from the ^{19}F OS-ENDOR spectra (*step 2*). Finally, the label was rotated around the Gd–F axis to minimize the distance between the sulfur atom of **Gd-DO3A** and the sulfur atom of the labeled cysteine residue (*step 3*).

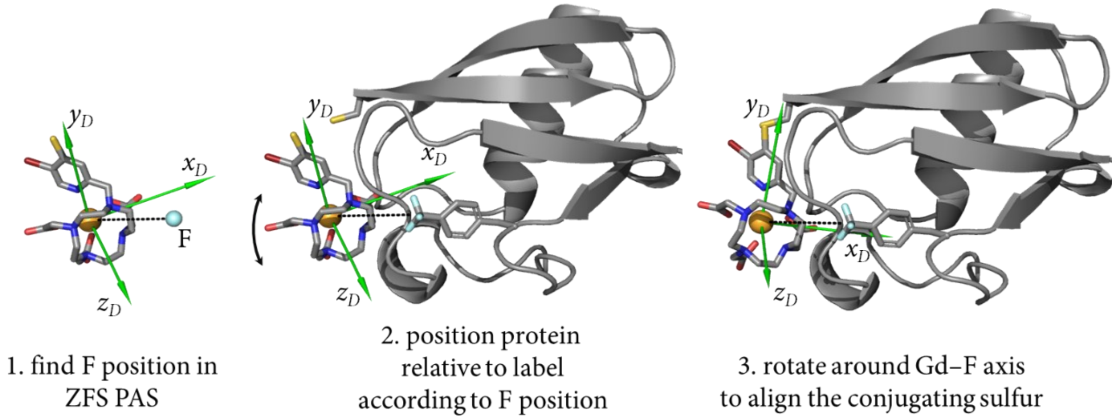

Fig. S18. Illustration of the molecular-modeling procedure for grafting the tag onto a protein based on experimental ^{19}F OS-ENDOR data.

In the 5F-Trp43 **GB1-Q32C** structure, a single fluorine atom is present, while in 4tFm-Phe45 **Ub-M1C,** three F atoms are present in the trifluoromethyl group. For the latter, an average position for the three fluorine atoms was used for the alignment.

The **Gd-DO3A** spin label exists as two enantiomers that interconvert in solution (for analogy, Gd-DOTA structural fragment possesses a C_4 rotation axis, coinciding with the NNNN-cyclen normal, and no mirror planes). Both enantiomers of the spin label were considered separately when grafting onto the protein structure.

For all the structures with the spin label attached, only those were kept, for which (a) the sulfur atoms of the label and the protein cysteine were sufficiently close, < 2 Å, and (b) no clashes between atoms of the protein and the label were present (with a distance threshold of 1 Å). Using these criteria, four structures were obtained for labeled 5F-Trp43 **GB1-Q32C**, shown in **Fig. S19**, and five structures were found for 4tFm-Phe45 **Ub-M1C**, shown in **Fig. S20** (a pair of representative structures is shown in **Fig. 8**, *main text*).

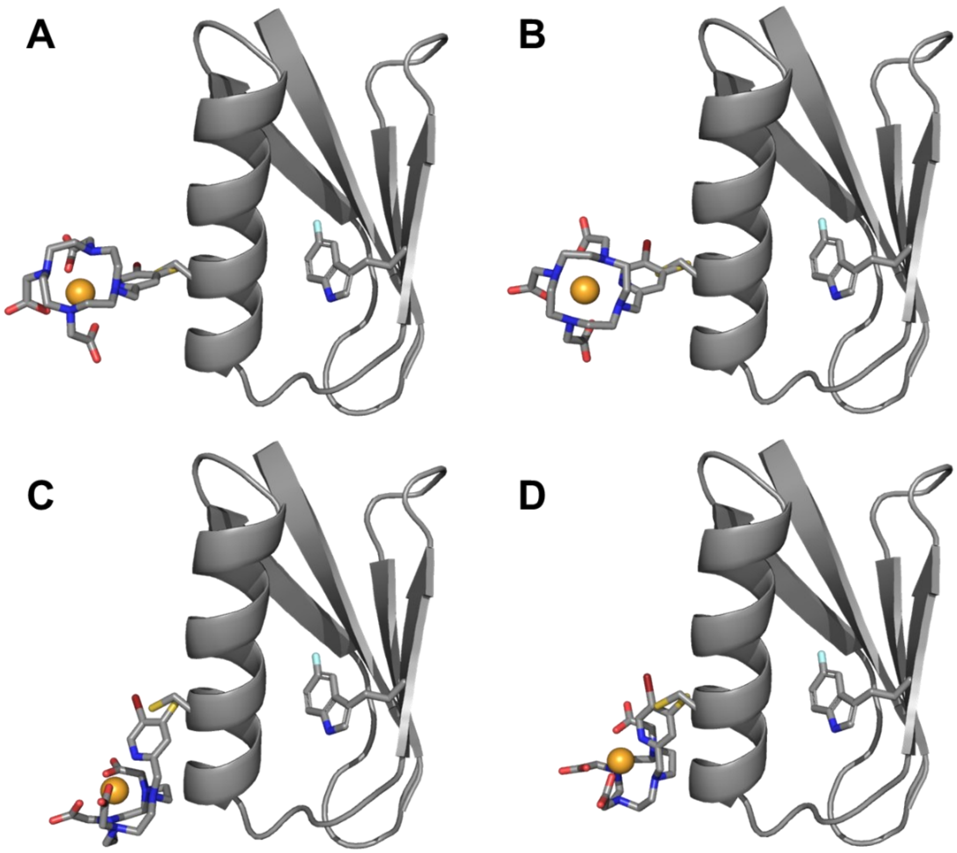

Fig. S19. Structures of **Gd-DO3A** labeled 5F-Trp43 **GB1-Q32C** based on ^{19}F OS-ENDOR spectra and imposed geometric constraints.

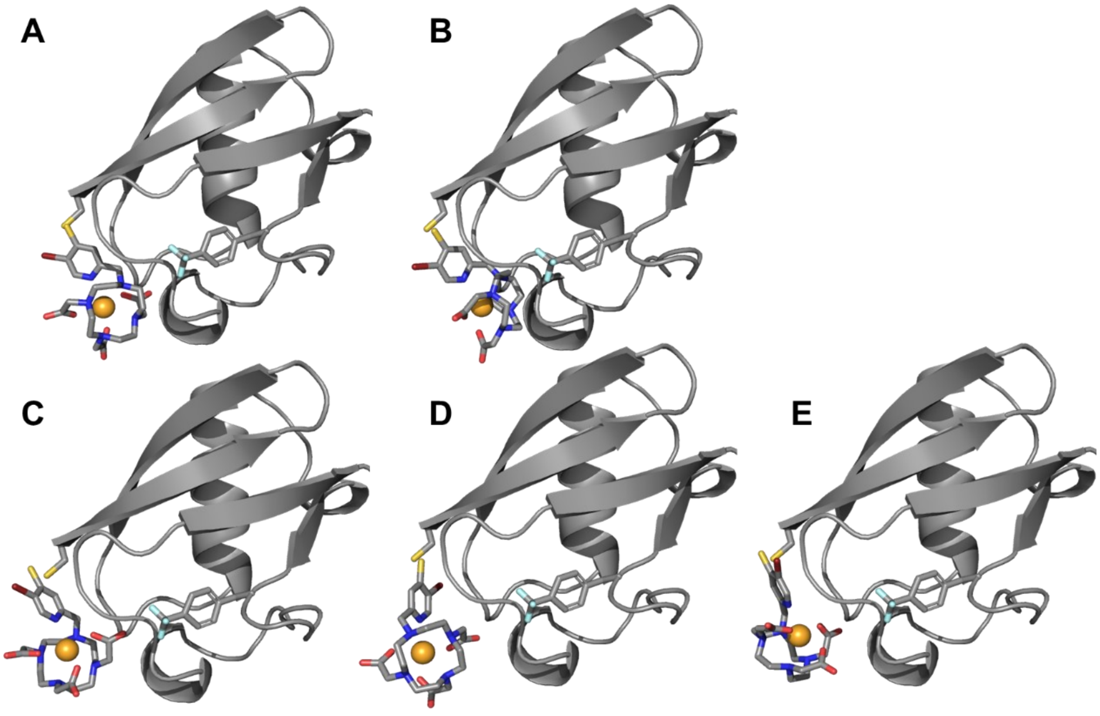

Fig. S20. Structures of **Gd-DO3A** labeled 4tFm-Phe45 **Ub-M1C**, based on ^{19}F OS-ENDOR spectra and imposed geometric constraints.

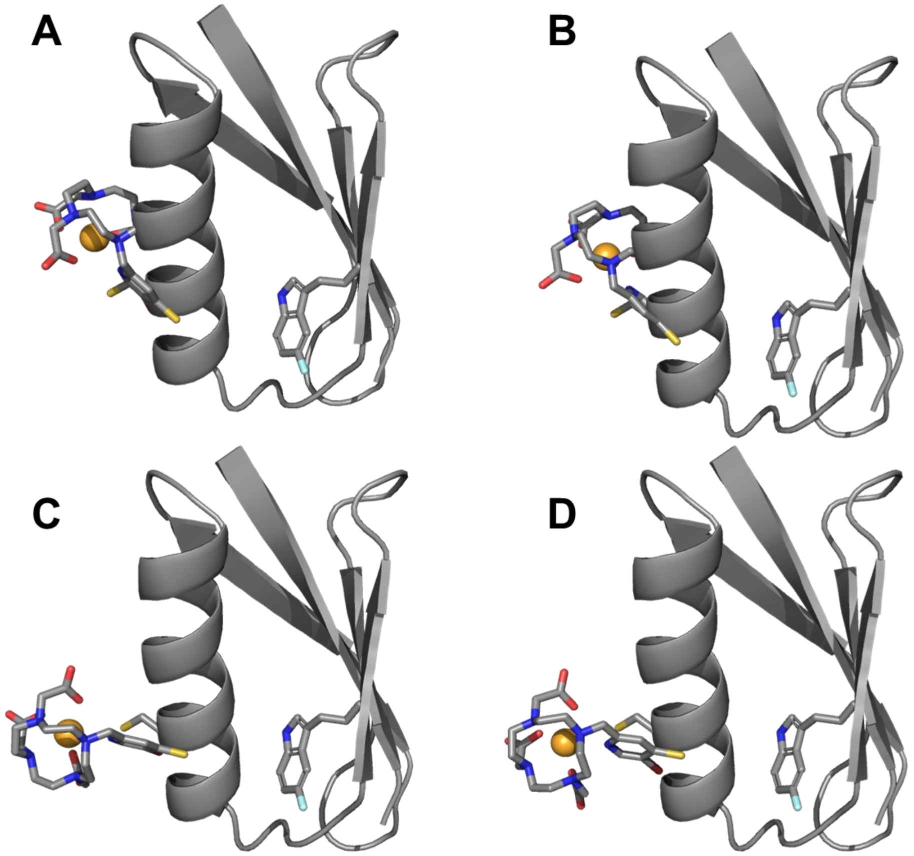

Fig. S21. Structures of **Gd-DO3A** labeled 5F-Trp43 **GB1-Q32C**, in which the fluorinated Trp43 residue (shown in stick representation) was flipped by 180º. The spin label was positioned and oriented based on the ^{19}F OS-ENDOR data. None of these structures were devoid of steric clashes and satisfied the distance restraint between the S-atoms of the cysteine and the label ($r_{SS} > 5$ Å for all found positions).

S11. Background subtraction of ^{19}F ENDOR spectra

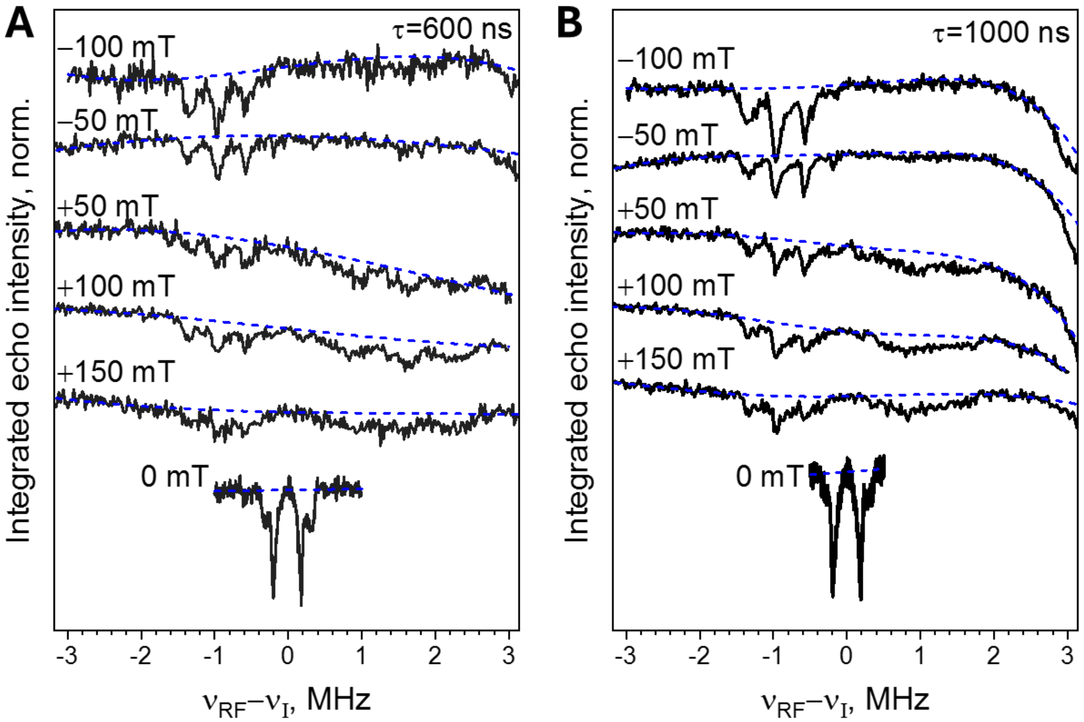

Fig. S22. Uncorrected ^{19}F OS-ENDOR spectra of **1** recorded using interpulse delays in the Mims sequence of τ=600 ns (**A**) and τ=1000 ns (**B**), respectively. Background subtraction was carried out using cubic or linear polynomials, depicted by the blue dashed lines. The baseline corrected spectra are shown in **Fig. 3A** in the main text. The increase of ENDOR intensity at higher RF is due to the overlapping ^{1}H ENDOR spectra corresponding to high-|m_S| electron transitions.

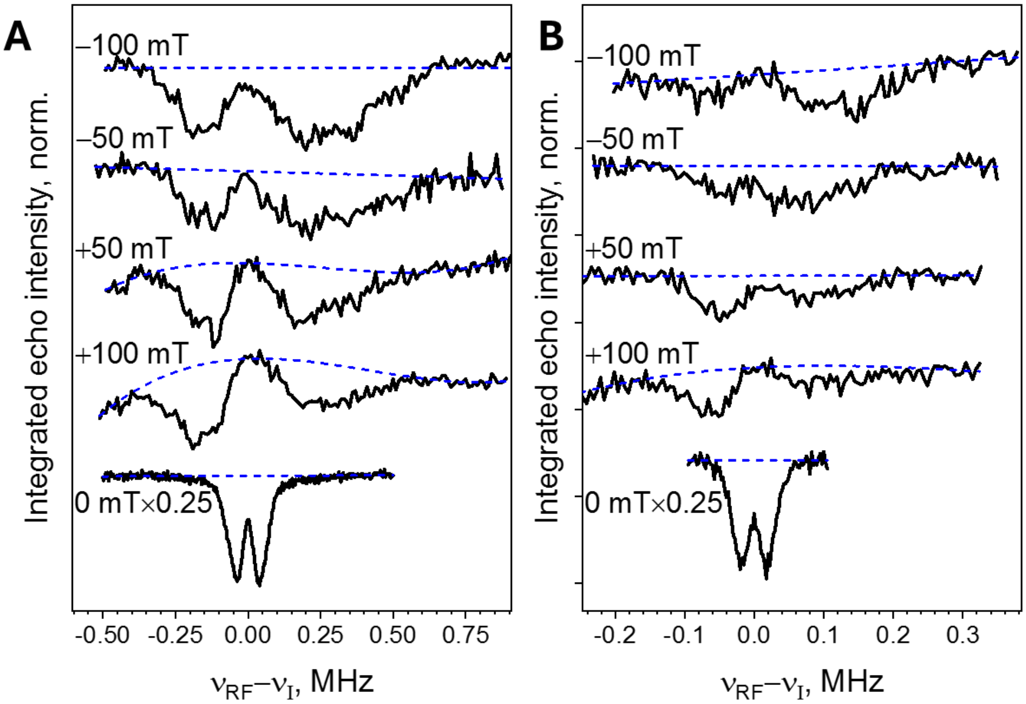

Fig. S23. Uncorrected ^{19}F OS-ENDOR spectra of **3** (**A**) and **4** (**B**). Backgrounds used for subtraction used cubic or linear polynomials, depicted by the blue dashed lines. The baseline corrected spectra are shown in **Fig. 3C, D** in the main text.

S12. Electron spin relaxation of the studied samples

Table S3. Electron spin phase memory times T_M and spin-lattice relaxation times T_{1e} for the studied systems. Corresponding stretched exponents are shown in parentheses. Uncertainties are of the order of ±5% for the time constants and of ±0.05 for the exponents.

**System**	**Comments**	**T_M, µs (β_M)**	**T_{1e}, µs (β_1)**
**1**	10 K	6.3 (1.3)	55 (0.65)
**2**	10 K	9.5 (1.2)	62 (0.62)
**3**	10 K	12.6 (1.4)	51 (0.65)
**4**	10 K	7.4 (1.4)	43 (0.64)
**GB1-Q32C Gd-DO3A**	10 K	6.7 (1.0)	45 (0.66)
**Ub-M1C Gd-DO3A**	10 K	5.4 (1.6)	53 (0.70)

Supplementary references

(S1) Neese, F. Software Update: The Orca Program System—Version 5.0. *WIREs Comput. Mol. Sci.* **2022**, *12* (5), e1606.

(S2) Perdew, J. P.; Ernzerhof, M.; Burke, K. Rationale for Mixing Exact Exchange with Density Functional Approximations. *J. Chem. Phys.* **1996**, *105* (22), 9982-9985. DOI: 10.1063/1.472933.

(S3) Grimme, S.; Ehrlich, S.; Goerigk, L. Effect of the Damping Function in Dispersion Corrected Density Functional Theory. *J. Comp. Chem.* **2011**, *32* (7), 1456-1465. DOI: 10.1002/jcc.21759.

(S4) Weigend, F.; Ahlrichs, R. Balanced Basis Sets of Split Valence, Triple Zeta Valence and Quadruple Zeta Valence Quality for H to Rn: Design and Assessment of Accuracy. *Phys. Chem. Chem. Phys.* **2005**, *7* (18), 3297-3305, 10.1039/B508541A. DOI: 10.1039/B508541A.

(S5) Cossi, M.; Barone, V. Analytical Second Derivatives of the Free Energy in Solution by Polarizable Continuum Models. *J. Chem. Phys.* **1998**, *109* (15), 6246-6254. DOI: 10.1063/1.477265.

(S6) Elamin, K.; Jansson, H.; Kittaka, S.; Swenson, J. Different Behavior of Water in Confined Solutions of High and Low Solute Concentrations. *Phys. Chem. Chem. Phys.* **2013**, *15* (42), 18437-18444, 10.1039/C3CP51786A. DOI: 10.1039/C3CP51786A.

(S7) Angarita, I.; Mazzobre, M. F.; Corti, H. R.; Longinotti, M. P. Revisiting the Glass Transition Temperature of Water–Glycerol Mixtures in the Bulk and Confined in Mesoporous Silica. *Phys. Chem. Chem. Phys.* **2021**, *23* (31), 17018-17025, 10.1039/D1CP02153B. DOI: 10.1039/D1CP02153B.

(S8) Bogdanov, A.; Frydman, V.; Seal, M.; Rapatskiy, L.; Schnegg, A.; Zhu, W.; Iron, M.; Gronenborn, A. M.; Goldfarb, D. Extending the Range of Distances Accessible by ^{19}F Electron–Nuclear Double Resonance in Proteins Using High-Spin Gd(III) Labels. *J. Am. Chem. Soc.* **2024**, *146* (9), 6157-6167. DOI: 10.1021/jacs.3c13745.

(S9) Bogdanov, A.; Seal, M.; Goren, E.; Bar-Shir, A.; Goldfarb, D. Host–Guest Geometry in Paramagnetic Cavitands Elucidated by ^{19}F Electron-Nuclear Double Resonance. *Phys. Chem. Chem. Phys.* **2025**, *27* (7), 3885-3896. DOI: 10.1039/D4CP04734F.

(S10) Gemperle, C.; Schweiger, A. Pulsed Electron-Nuclear Double Resonance Methodology. *Chem. Rev.* **1991**, *91* (7), 1481-1505. DOI: 10.1021/cr00007a011.

(S11) Dennis Jr, J. E.; Gay, D. M.; Walsh, R. E. An Adaptive Nonlinear Least-Squares Algorithm. *ACM Transactions on Mathematical Software (TOMS)* **1981**, *7* (3), 348-368. DOI: 10.1145/355958.355965.

(S12) Schrödinger, L. *The Pymol Molecular Graphics System, Version 2.5.3*; http://www.pymol.org.

(S13) Kabsch, W. A Solution for the Best Rotation to Relate Two Sets of Vectors. *Acta Crystallogr., Sect. A* **1976**, *32* (5), 922-923. DOI: 10.1107/S0567739476001873.

(S14) Angeli, C.; Cimiraglia, R.; Evangelisti, S.; Leininger, T.; Malrieu, J.-P. Introduction of N-Electron Valence States for Multireference Perturbation Theory. *J. Chem. Phys.* **2001**, *114* (23), 10252-10264. DOI: 10.1063/1.1361246 (acccessed 2/24/2026).

(S15) Pollak, P.; Weigend, F. Segmented Contracted Error-Consistent Basis Sets of Double- and Triple-Z Valence Quality for One- and Two-Component Relativistic

All-Electron Calculations. *J. Chem. Theory Comput.* **2017**, *13* (8), 3696-3705. DOI: 10.1021/acs.jctc.7b00593.
(S16) Hagelueken, G.; Abdullin, D.; Schiemann, O. Mtsslsuite: Probing Biomolecular Conformation by Spin-Labeling Studies. In *Methods in Enzymology*, Vol. 563; Elsevier, 2015; pp 595-622.
(S17) Horn, B. K. Closed-Form Solution of Absolute Orientation Using Unit Quaternions. *J. Opt. Soc. Am. A* **1987**, *4* (4), 629-642. DOI: 10.1364/JOSAA.5.001127.
(S18) Jeschke, G. Mmm: A Toolbox for Integrative Structure Modeling. *Protein Sci.* **2018**, *27* (1), 76-85. DOI: 10.1002/pro.3269.
(S19) Gronenborn, A. M.; Filpula, D. R.; Essig, N. Z.; Achari, A.; Whitlow, M.; Wingfield, P. T.; Clore, G. M. A Novel, Highly Stable Fold of the Immunoglobulin Binding Domain of Streptococcal Protein G. *Science* **1991**, *253* (5020), 657-661. DOI: 10.1126/science.1871600.
(S20) Vijay-Kumar, S.; Bugg, C. E.; Cook, W. J. Structure of Ubiquitin Refined at 1.8 Å Resolution. *J. Mol. Biol.* **1987**, *194* (3), 531-544. DOI: 10.1016/0022-2836(87)90679-6.
(S21) Seal, M.; Zhu, W.; Dalaloyan, A.; Feintuch, A.; Bogdanov, A.; Frydman, V.; Su, X.-C.; Gronenborn, A. M.; Goldfarb, D. Gd^{III}-^{19}F Distance Measurements for Proteins in Cells by Electron-Nuclear Double Resonance. *Angew. Chem., Int. Ed.* **2023**, *62* (20), e202218780. DOI: 10.1002/anie.202218780.
